# Categorical colour geometry

**DOI:** 10.1371/journal.pone.0216296

**Published:** 2019-05-10

**Authors:** Lewis D. Griffin, Dimitris Mylonas

**Affiliations:** Computer Science, UCL, London, United Kingdom; Lancaster University, UNITED KINGDOM

## Abstract

Ordinary language users group colours into categories that they refer to by a name e.g. *pale green*. Data on the colour categories of English speakers was collected using online crowd sourcing – 1,000 subjects produced 20,000 unconstrained names for 600 colour stimuli. From this data, using the framework of Information Geometry, a Riemannian metric was computed throughout the RGB cube. This is the first colour metric to have been computed from colour categorization data. In this categorical metric the distance between two close colours is determined by the difference in the distribution of names that the subject population applied to them. This contrasts with previous colour metrics which have been driven by stimulus discriminability, or acceptability of a colour match. The categorical metric is analysed and shown to be clearly different from discriminability-based metrics. Natural units of categorical length, area and volume are derived. These allow a count to be made of the number of categorically-distinct regions of categorically-similar colours that fit within colour space. Our analysis estimates that 27 such regions fit within the RGB cube, which agrees well with a previous estimate of 30 colours that can be identified by name by untrained subjects.

## 1. Introduction

Colour is a perceptual quality sitting midway in a causal chain linking the physical world (light sources, surfaces, sensory mechanisms) to the cultural (categories, concepts, language). The entire chain is active when a subject views (say) a leaf, and declares it to be ‘green’. In this paper we use colour categories to define a geometry on the manifold of surface colours. Colours are close in this geometry if they are categorized similarly.

In the introduction we review previous work on colour geometry, on colour categories, and on the relation between colour categories and geometry. In section 2 we review Information Geometry, which is the mathematical framework we use to derive the categorical geometry, and apply it to a toy model of colour space and its categories. In section 3 we describe the collection of the colour naming data on which we base the categorical geometry. In section 4 we characterize the naming dataset. In 5 we use the dataset to constrain a continuous model of naming across colour space. In 6 we compute the categorical metric from that model. In 7 we analyse the resulting geometry, and in 8 we discuss.

### 1.1 Colour spaces

The sensory mechanisms of colour vision are well understood [1]. Three classes of retinal cone compute linear functions of the spectral power of the retinal irradiance (*I*), yielding a triple of scalar responses i.e. $\langle l,m,s\rangle =\int_{\lambda \in \left[380,750\right]}\langle L\left(\lambda \right),M\left(\lambda \right),S\left(\lambda \right)\rangle I\left(\lambda \right)$. Since both spectral power and cone sensitivity are non-negative, and the sensitivity functions overlap, not all sensory triples are possible. The possible triples lie within a convex generalized cone known as the *spectral cone*. Its tip is the response 〈0,0,0〉 to no light. Each half-ray of its curved boundary are the responses to a particular monochromatic light (of varying power) or to a particular mixture of monochromatic lights from both extremes of the visible range. Other lights give responses in the cone interior. Luminous colours are considered to be one-to-one with sensory responses in and on the spectral cone.

A different treatment is needed for surface colours because, for example, brown is a surface but not a luminous colour [2]. Surface colour arises from an interaction between the illuminant and the spectral reflectance of the surface. Specifically, the light reflected from a Lambertian surface has no more energy at each wavelength than the illuminant. This leads to a restriction on the sensory responses that can arise from illuminated surfaces, given a fixed illuminant. The restricted set of responses is a convex sub-region (roughly the shape of a rugby ball) of cone response space known as the *body colour solid* [3]. The colour solid lies in the interior of the spectral cone, apart from the black response 〈0,0,0〉 which is the tip of the spectral cone and one of the two sharp points of the solid, the other being the white point. Surface colours are considered to be one-to-one with sensory responses in and on the colour solid.

**Fig 1:**
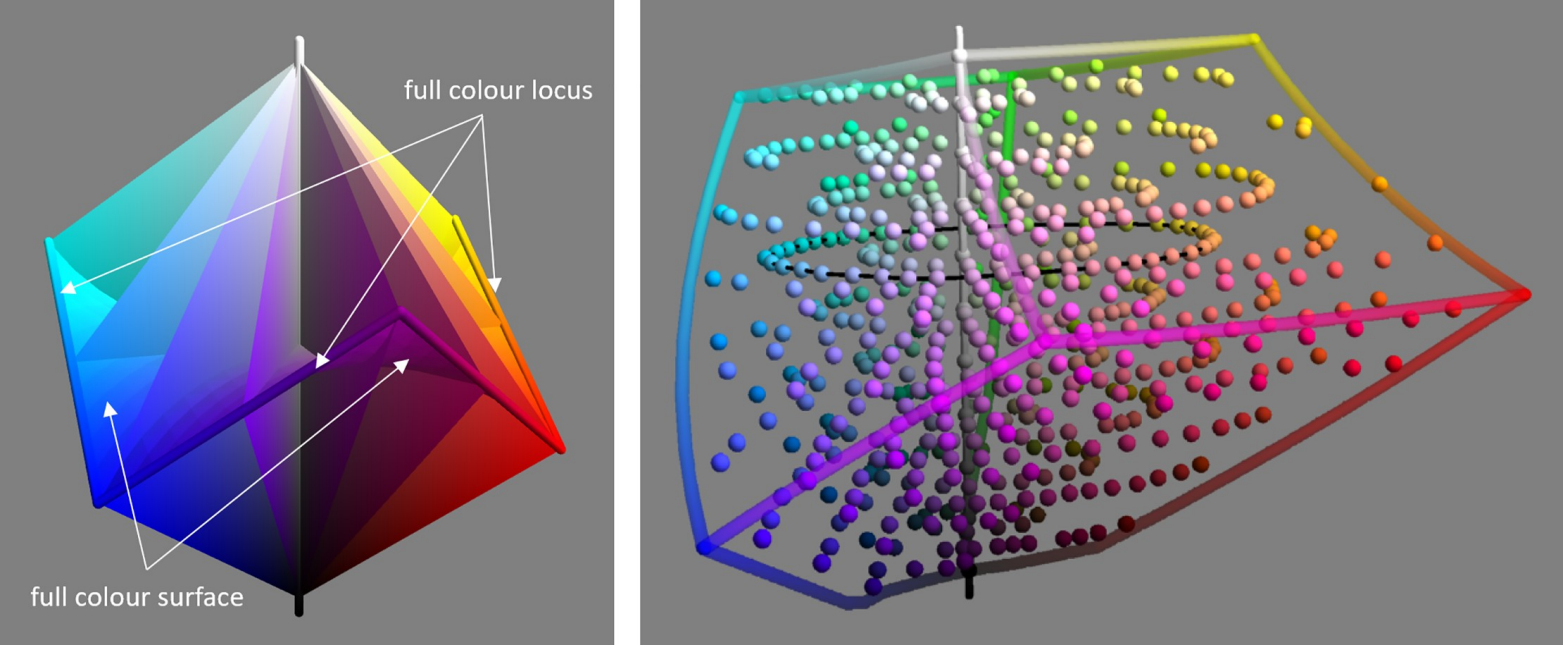
Illustrates the sRGB (left) and CIELAB (right) colour spaces. Left: the achromatic axis (vertical) and the full colour locus (non-planar hexagon) are marked by tubes. A selection of hue triangles span from the achromatic axis to the full colour locus. The curved full colour surface, bordered by the full colour locus, can just been seen. Right: the extent of the sRGB cube is shown in CIELAB space. The colours for which naming data was collected (section 2) are shown by spheres. The thin black horizontal ring marks a path which is analysed in section 5 Fig 8.

Cone response triples can be used as a coordinate system for colours, as can linear transformations of them e.g. 〈*X*,*Y*,*Z*〉 [4]. Several different coordinate systems, that are non-linear functions of the cone responses, have been devised for the colour solid. In this work we make use of CIELAB [5] and sRGB [6] (Fig 1). An advantage of sRGB coordinates is that the extent of the colour solid is well approximated as a cubical region i.e. 〈*R*,*G*,*B*〉∈[0,1]^3^ [7], whereas the shape is much more complicated to capture in CIELAB. An advantage of CIELAB is that Euclidean distances better approximate JND step distances than they do in sRGB.

### 1.2 Colour geometry

The space of lights (described by their spectral power) has a vector space structure with addition corresponding to optical mixing. Colour space inherits this vector space structure because of the linear operation of the cones, with the dimensionality dropping from infinite to three [7]. A vector space does provide *some* geometrical structure. It *does* allows colours to be scaled and added; colour differences to be compared, and judged parallel or not; and parallel colour differences to be compared in magnitude; but it does *not* allow comparison of arbitrary differences. This has spurred the search for a richer colour geometry.

While a full colour metric has been the aim of most research [8, 9], there are interesting geometries that are weaker than metrical, for example based on ‘between-ness’ [10]. Towards such a geometry, subjective judgements have been used to construct lines of constant hue [11], and lines between arbitrary colour endpoints [12]. These lines are typically curved relative to the vector space structure.

The idea of a colour metric seems to have arisen first in *mathematics*, in Riemann’s seminal essay in which he generalized the idea of metrical structure to encompass spaces of smoothly varying geometry [13]. That essay opens by reviewing what the author says are the only two continuous multi-dimensional manifolds of common experience: physical space and colour space. The (Riemannian) manifolds it goes on to define are everywhere locally Euclidean in the infinitesimal limit but are ‘glued’ together in such a way to permit varying geometry. The machinery needed to express this variable geometry is a metric tensor at each location that expresses the distortion between a local coordinate chart and the space described. The metric tensor permits the length of any infinitesimal displacement through the location to be determined. Given the metric tensors, the length of a path can be defined through integration, and the distance between two locations can be defined as the length of the shortest path (geodesic) connecting them.

Riemann’s work showed that an inhomogeneous geometrical structure is coherent and how to specify it, but it is silent on what determines the metric for any particular manifold, including colour. Helmholtz seems to have been the first to consider a colour metric grounded on the *discriminability* of colours. This construction relies on the stochastic nature of discriminability – as the separation between two colours is increased, the *proportion* of trials in which a subject would judge them as different increases. A just-noticeable-difference (JND) can be defined from this pattern, most commonly by defining it as the separation such that inequality is correct distinguished from equality 75% of the time [14]. JNDs provide a portable unit of distance, applicable anywhere in colour space. A metric tensor field can be defined which measures lengths in the common currency of JNDs.

Helmholtz proposed equations for the colour metric tensor, as functions of sensory response coordinates, that he hoped would match colour discriminability [15]. Various authors (including Schrodinger [3]) proposed alternative formula, all attempting to account for scant colour discriminability data. Finally MacAdam [16] published a definitive dataset, based on laborious experiments, of JND-based metric tensors at many points of luminous colour space. More recently discrimination data has been modelled as deriving purely from receptor noise [17–19].

MacAdam’s metric is a systemization of facts about when two *luminous* colours can be seen to be different. Many practical applications are more concerned with discriminability of *surface* colours, and a different tradition of practitioners have established these, most famously Munsell [20]. Munsell spaced the colour samples in his atlas using pairwise comparison and adjustment e.g. choose *a* to equalize the magnitudes of the colour differences *ab* and *cd*. Munsell devised a web of paired comparisons to propagate the same unit of difference throughout the colour solid [21]. The Munsell unit of difference is small enough that it is assumed to correspond to the same small number of JNDs wherever it is established.

Munsell’s metrical results apply to physical coloured samples, but can be converted to a metric over the colour solid. The CIE have specified metrics for the colour solid directly in terms of sensory coordinates. They choose to do this by specifying an ‘approximately uniform’ colour space, with coordinates 〈*L*,*a*,*b*〉 continuously dependent on sensory responses, in which Euclidean distance approximates the minimal number of JND steps between two colours. It is an empirical matter whether the JND structure of colour space allows it to be isometrically embedded in 3-D Euclidean space in this way. New non-Euclidean distance formulas (CIE1994 [22, 23] and CIE2000 [24]) have been specified in terms of CIELAB coordinates. At a statistical level these new distances fit discrimination and equal-step data better, but they do not always satisfy the triangle inequality (*d*_*ac*_≤*d*_*ab*_+*d*_*bc*_) and so, strictly speaking, do not define a metric.

Similarity and discriminability are equal in the limit as colour separation goes to *zero*, and it is reasonable to assume that similarity is proportional to JNDs steps for *small* colour differences, but it is doubtful that it does so for *large* colour differences – it certainly does not for all sensory continua. For example, in the domain of pattern regularity, patterns which are judged equally different from both perfect regularity and from full randomness are 5.4 JNDs (of regularity level) from randomness and 11.1 JNDs from perfect regularity (Fig 12 in [25]). See also the discussion of categorical perception in section 1.3. Regardless of their deviation from discriminability, various types of judgment of large colour differences have been used. For example, *ratings* (‘how similar are *a* and *b* on a scale of 1 to 5?’) as input to multi-dimensional scaling [26]; *pairwise comparison*s (‘*ab* or *ac*?’) to test for a Euclidean metric [27]; and *midpoint* settings (‘choose *c* so that it is equally and maximally similar to *a* and *b*’) to test for affine structure [28].

Recently a very different metric for surface colour has been proposed that is grounded, not on JNDs, but on colours being ‘vaguely’ the same [29]. This definition is operationalized by having subjects adjust a surface colour so that it matches a simultaneously present, but not contiguous, target colour. The unit of ‘vaguely the same’ is portable just as JNDs but is 10-20 times larger. It is unclear to what extent this new metric has a different intrinsic structure to JND based ones.

### 1.3 Colour categories

English (and similarly other languages) has colour *names* (e.g. ‘red’, ‘peach’, ‘warm brown’) that refer to a (possibly fuzzy) region of colour space known as a colour *category*; the category forms part of the *concept* related to the name, plus other information such as what objects typically have that colour.

Treating modified colour names (e.g. light pink) as distinct from unmodified, counts of the number of English colour names reach into the thousands [30], but individual speakers make use of only ~30 colour names in ordinary usage [31], and analysis of category overlap suggests that there are only ~50 distinct categorical territories in colour space [32].

Some colour names are used more frequently and consistently than others, this has been captured in the idea of a subset of terms having a special *basic* status, originally defined by a combination of linguistic and psychological criteria [33]. In English the 11 Basic Colours are considered to be black, grey, white, red, orange, yellow, green, blue, purple, pink and brown. More objective criteria for basicness have been advanced [34–36], for example that they are indispensable for naming some colours [37]; and the case has been made that basicness is more of a graded scale than a dichotomy [38, 39].

Colour categories have been mapped in many languages, most systematically in the World Color Survey [40] and in online data collection [41]. This has shown that there are both differences [42, 43] and commonalities across languages in terms of the numbers of categories and the positioning of their borders within color space [44]. A considerable literature has considered whether this data speaks for [45, 46] or against [33, 47] the Sapir-Whorf hypothesis that language determines categories (and categories influence perception and thought) [48]; and what, if any, non-linguistic factors shape colour categories. Contenders for non-linguistic influences being: environmental regularities [49], variations in stability under illuminant change [50], neural coding alignments [51], and utility for object recognition [52]. A compromise conclusion, which reconciles the evidence, is that ‘nature proposes certain universal constraints, and nurture disposes of these constrains depending on culture-specific learning [53].

### 1.4 Categories & geometry

Wittgenstein, searching for a logic of colour concepts, asserted as self-evident that ‘Yellow is more akin to red than to blue’ [54]. This is a geometrical fact about colour categories. Such statements turn out to be pretty consistent at the population level [55], and have been shown to mirror the shape of the body colour solid. Whether they are in line with (say) JND geodesic distances between category foci, or whether they follow an independent logic is unknown.

A second way in which categories and geometry interact is in the phenomenon of *categorical perception*. The phenomenon, first observed for other sensory continua [56], concerns when similarity judgments between well-separated colours fail to agree with JND separation (or when JND separation fails to agree with sensory coding distance [57]) depending on whether the colours involved are within or across categories. In particular apparent distance can be increased when colours belong to different categories, and reduced when they belong to the same [58, 59]. Although issues have been raised concerning the methodology used [60], recent results showing the effects in non-humans lend additional support [61].

The name – categorical perception – pre-supposes an explanation for it, that the category of the colour is manifest in the perception of it; an alternative explanation for the phenomenon is that categories become relevant when the colour difference is assessed [39, 62]. Regardless of the explanation, the phenomenon rules out a single Riemannian model that accounts for both colour discrimination and similarity judgments. Potentially judgments involving only large differences are compatible with a Riemannian metric, but different from the one that accounts for discriminations and small difference judgments. Or possibly a non-Riemannian geometrical structure needs to be devised that could account for all judgements within a single framework.

## 2 Information geometry

In this paper we use the framework of Information Geometry [63] to compute a Riemannian geometry for colour based on naming data. In this section we review Information Geometry and then specialize it to colour naming in worked examples.

Information Geometry is concerned with defining a metric on statistical manifolds, which are continuous multi-dimensional families of probability distribution functions (pdfs). It defines the metric by considering how well samples drawn from one of those distributions allow one to determine which distribution was the source. More formally, the metric is an infinitesimal generalization of the Jensen Shannon Distance (JSD) [64].

We define the JSD between distributions *P* and *Q* both over a domain Ω. Suppose samples *x* are drawn with equal probability from either *P* or *Q*; and consider how much information, on average, *x* carries about what its source was. If *P* and *Q* are very different then often the source of *x* will be unambiguous and so the JSD will be near its maximum value. On other hand if *P* and *Q* are very similar then mostly *x* will just tilt the odds slightly in favour of *P* or *Q* and so the JSD will be small. The symmetric JSD can be succinctly specified in terms of the non-symmetric Kullback-Lieber Divergence (KLD) and the mixture distribution *M*≔(*P*+*Q*)/2 as follows:

$$\begin{array}{l} d_{JS}\left(P,Q\right)≔\sqrt{\frac{1}{2}\left(D_{KL}\left(P,M\right)+D_{KL}\left(Q,M\right)\right)}\\ D_{KL}\left(X,Y\right)≔-\int_{\Omega }X\log_{2}\left(Y/X\right) \end{array}$$

The information metric of a statistical manifold is based on measuring the infinitesimal JSD between ‘neighbouring’ distributions which are only infinitesimally different. The length of a path through a statistical manifold can then be defined by integration of these infinitesimal distances.

Consider a specific example, the manifold of univariate normal distributions indexed by mean and standard deviation $\langle \mu ,\sigma \rangle \in \mathbb{R}\times {\mathbb{R}}^{+}$. The infinitesimal distance between the distributions indexed by 〈*μ*,*σ*〉 and 〈*μ*+*δμ*, *σ*+*δσ*〉 is $\delta s=\sqrt{\frac{1}{4}\delta \mu^{2}+\frac{1}{2}\delta \sigma^{2}}/\sigma$. So the information distance from 〈0,1〉 to 〈*ε*,1〉 is ε2; which is the same as the distance to $\langle 0,1+\varepsilon /\sqrt{2}\rangle$. Normally the distance is written as $\delta s^{2}={\left(\begin{array}{c} \delta \mu \\ \delta \sigma \end{array}\right)}^{T}\left(\begin{array}{cc} \frac{1}{4}\sigma^{-2} & 0\\ 0 & \frac{1}{2}\sigma^{-2} \end{array}\right)\left(\begin{array}{c} \delta \mu \\ \delta \sigma \end{array}\right)$; where the 2×2 metric tensor is called the Fisher information matrix. Note that while the metric tensor in this example is diagonal, this is not required.

The exact same Fisher information metric can be derived in other ways than via the JSD. For example as the second derivative of the KLD, or in terms of the first derivatives of the logarithms of the pdfs. We consider that a particularly intuitive formulation, which also lends itself to numerical computation, is in terms of square-rooted pdfs. Square-rooted pdfs have unit L^2^ norm (because pdfs have unit integral), so can be considered as points on the surface of a unit hypersphere of appropriate dimension. Information distance is geodesic distance on that hypersphere, which is equal to the angle subtended from the sphere centre. This is known as the Bhattacharya angle and can be computed by $d_{bhatt}\left(P,Q\right)≔\arccos\left(\int_{\Omega }\sqrt{PQ}\right)$. If the distributions are sufficiently close, then this is just the Euclidean distance i.e. $d_{bhatt}\left(P,P+\delta P\right)\approx \left\|\sqrt{P}-\sqrt{P+\delta P}\right\|$.

Although information geometry is typically used to study the structure of a statistical manifold, whose points *are* distributions, it can also be used to equip a manifold (of something other than distributions) with a metric *if* there is a distribution associated with each manifold point. This is the way we use it here: our concern is with the colour manifold augmented with the distributions of names evoked by each colour. Information geometry will allow us to define a metric on the colour manifold from the variation in these distributions of names. We call that the *categorical metric*. In the categorical metric, colours which are named similarly will be close together; while colours which are named differently will be far apart. The distances will be entirely driven by the pattern of naming, and need not correlate with JND distances. We work through an example to develop familiarity.

### 2.1 Worked example

**Fig 2:**
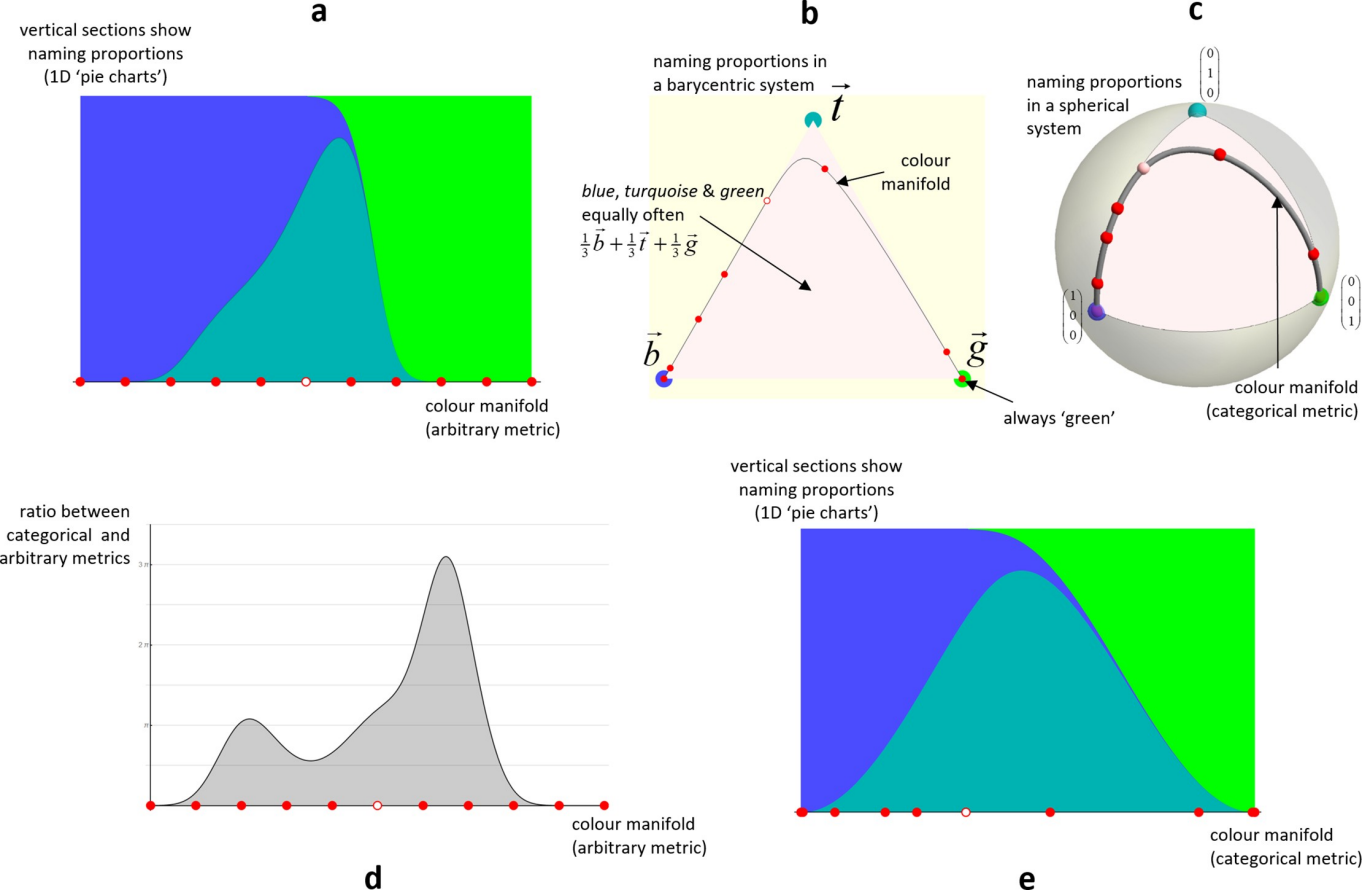
A toy colour naming example. **a**) Plots the distribution of naming responses (vertical) for different locations (horizontal) in a hypothetical 1-D colour manifold. Red points mark points in the colour manifold which can be followed across the panels. **b**) A replot of the previous panel, with location within the triangle indicating the distribution of naming responses. **c**) A replot of the previous panel, with location on the sphere indicating the distribution. **d**) Same horizontal scale as **a**, the vertical scale indicates the categorical distance (determined by the change in naming responses) for a small change in colour at that location in the colour manifold. **e**) A warp of **a** so that horizontal distances in the figure equal categorical distances.

Fig 2A visualizes a hypothetical 1-D colour manifold whose colours only ever evoke three *chromatic* names (say *blue*, *turquoise* and *green*) in different proportions. So the colour plotted at the left is always named *blue*, intermediate colours evoke all three names in varying proportion, and the colour at the right is always named *green*. Note that the manifold is plotted with an arbitrary embedding into Euclidean space; any continuous warp of the diagram would be just as valid, it would translate the vertical sections but not change them. With that noted, we draw attention to the tick marks along the horizontal axis – we will follow these through the sequence of plots in the figure.

In **b** we have replotted the manifold using a barycentric coordinate system [65] based on a triangle of points in the plane with locations $\vec{b},\vec{t},\vec{g}\in E^{2}$ corresponding to response distributions which are 100% one particular name. The response distribution with probability values 〈*b*,*t*,*g*〉 (hence *b*+*t*+*g* = 1) is plotted at $b\vec{b}+t\vec{t}+g\vec{g}$. The marks along the curve of **b** correspond to the marks of **a**. Observe how their spacing has changed from **a** to **b**, and how several ticks at the left and right extremes of **a** are collapsed onto the $\vec{b},\vec{g}$ vertices of **b**. The manifold reaches the $\vec{b},\vec{g}$ vertices since there are colours with always *blue* and always *green* responses, but goes only close to $\vec{t}$ since no colour evokes *turquoise* only.

In **c** we have changed to square-rooted pdfs over the three colour names (i.e. bb→+tt→+gg→→(b12t12g12)T), these correspond to points on the unit 2-sphere (because ‖(b12t12g12)T‖2=b+t+g=1). The planar triangle in **b** maps to a spherical octant triangle in **c**. Observe how the mark spacing changes from **b** to **c**, and note how the curve does not go as tightly into the orange corner in **c** as it does in **b**. This is due to the square-rooting and reflects that a change in the response rate of a name for a colour from 5% to 10% is more easily noticeable on the basis of samples than a change from 45% to 50%. The manifold distances in this plot are categorical distances.

In **d** we use the same arbitrary embedding of the manifold as in **a**. The height of the curve shows the categorical distance between nearby colours (i.e. the speed in **c**). Observe how there is greater categorical distance the faster the response pattern is changing, particularly when elements of it are changing near zero.

In **e** we show an embedding of the manifold into Euclidean space that preserves categorical distances. The figure is just a warp of **a**, and **d** is the derivative of the warp. Observe how the regions of constant response distribution at the left and right extremes of **a** have shrunk to points in **e**, and how the region right-of-centre where the *turquoise*/*green* proportions change steeply, and a thin tail of *blue* gradually reduces, has been greatly expanded in **d**. As this figure is categorically isometric, any two pairs of equally-spaced nearby points have the same distinguishability of their response distributions.

The total categorical length of the manifold is its length on the unit radius 2-sphere in **c**, which is 2.7 units. This length is more clearly expressed in terms of *grains*, which are a natural unit of categorical extent corresponding to *the separation between two distributions without responses in common*. Each triangle edge in **c** is one grain long, and has a length of π2. So the categorical extent of the manifold is 2.7=1.7×π2=1.7grains. This is a bit less than two grains because (in this toy example) there are colours for which both *blue* and *green* responses occur, despite the *turquoise* category; had there been colours with always *turquoise* responses the extent would have been two grains.

Also worth noting is that although the response distributions (100% *blue* and 100% *green*) for the extremes of the manifold are completely disjoint, the distance between them is more than just one grain. This is because categorical distances are integrated infinitesimal distances, taking into account the sequences of changes along the path between two colours. This gives potential for the categorical metric to be compatible with, for example, Wittgenstein’s introspection that ‘red is more akin to yellow than blue’ [54].

The length of 1.7 grains can also be considered as an extent. From this perspective it should tell us how many categorically distinct *regions* of colour there are. However 1.7 grains does not imply 1.7 regions, as the domains at the ends of the manifold need only half normal extent (discussed in more detail in section 7.4). Hence an extent of 1.7 implies a *capacity* of 1.7+2×0.5=2.7 categorically distinct colours, which seems about right for this example.

**Fig 3:**
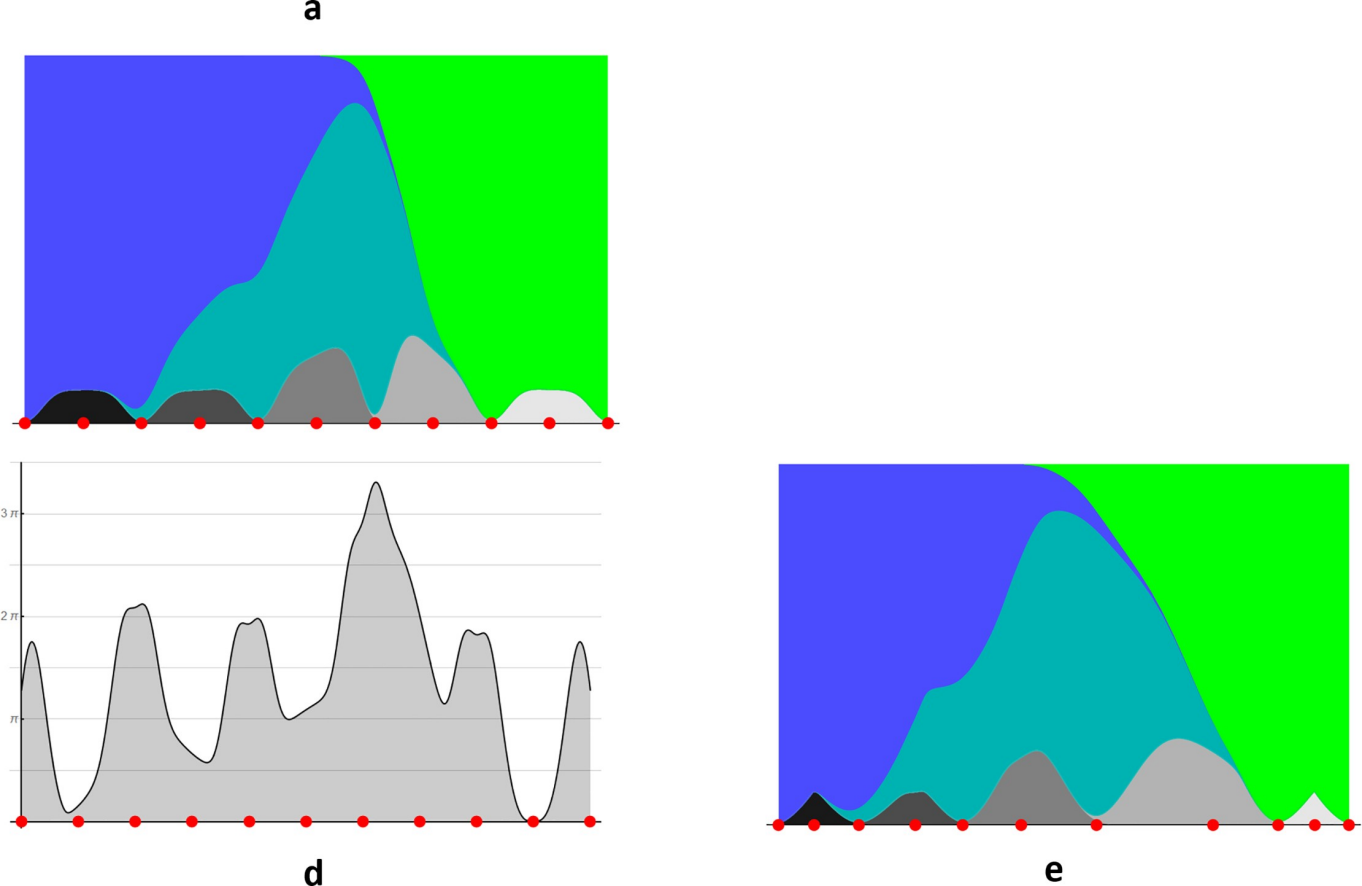
Same layout as Fig 2 but with a more complex pattern of naming responses. Panels **b** and **c** are omitted as too high-dimensional for visualization.

We extend the worked example in Fig 3 by addition of five extra *tonal* responses, with little overlap in their support and low peak response rates. Panels in Figs 2 and 3 correspond. Comparing the **d** panels, new humps have been added at the transitions of the *tonal* categories. The addition of these humps reduces the overall variability of the **d** curve. Specifically, for Fig 2 the standard deviation of the log_2_ of the metric is 4.9, for Fig 3 it is 1.7. The consequence of this reduced variability is that the isometric embedding in **e** is a milder warp of **a** in Fig 3 than in Fig 2. This is because the equally-spaced (in the arbitrary metric of **a**) *tonal* categories makes the arbitrary metric closer to the categorical one.

We can compare the capacities of the manifold with different systems of categories: *chromatic* is 2.7 regions; *chromatic* and *tonal* is 3.7 regions; *tonal* is 5.0 regions. Observe how addition of the low response rate *tonal* categories increases the capacity of the manifold but not to the size it would be with the *tonals* alone. It shows that the categorical metric arises as an interplay between category extents, overlaps and weights.

## 3. Collection of naming data

We used an online experiment to collect unconstrained colour naming responses to patches of colour (http://colournaming.org) presented on subjects’ own computers. Participation was voluntary and anonymous, and the experimental sessions were conducted after obtaining informed consent. To minimize the number of disruptive and poorly motivated participants a high entrance barrier format [66] was used. This required participants to provide metadata on colour deficiency, hardware/software components and viewing conditions before they then named sequentially presented colour patches.

The colours of the patches viewed by each subject were drawn randomly from a pool of six hundred selected from the Munsell Renotation dataset [67], including eleven achromatic samples. We call these samples *chips* though they are not physical. Chips were selected to be roughly perceptually uniform and covering the full colour gamut [41]. Fig 1 shows the chips plotted in CIELAB along with the extent of the sRGB cube, which we treat as a convenient approximation of the body colour solid. The figure shows that the chips are reasonably uniform in the approximately perceptual uniform CIELAB space, and fill up the sRGB cube.

- In the remainder of the paper we will use variables $\vec{c},\vec{z}$, both encoded in CIELAB coordinates, to range over the chips and the extent of the sRGB cube respectively.

Chips were specified in the sRGB standard colour space for the Internet, and rendered using each subject’s software drivers and display. They were presented as 147×94 pixel landscape rectangles on a neutral grey background with a black outline of one pixel. The grey background is sufficient for the colours be perceived in surface mode, so names such as grey and brown can be applicable. Subjects could type in any colour descriptor, either a single or multiple words, without time limit. Each named 20 chips sequentially presented. The 18^th^ chip presented was a repeat of the 3^rd^.

Data was collected over the period 2008-15 from unpaid subjects who visited the website and who declared themselves as English language speakers resident in the UK or US. Analysis of IP addresses confirmed geographical location in over 90% of cases; and 80% of subjects described themselves as ‘native speakers’, 1% as ‘bilingual’, 11% as ‘advanced’, 6% as ‘intermediate’ and 2% as ‘beginners’. Subjects were screened with an online version of the City Colour Vision Deficiency test, and 11% of subjects were excluded as possibly not colour vision normal. After exclusion, data was collected from 541 UK subjects and 525 US, yielding a total of 20,254 responses.

Based on this dataset, the inter-subject agreement rate (i.e. probability of obtaining the exact same response to a random chip) was 15%. Based on the repeat presentation of one chip for each subject (on the 3^rd^ and 18^th^ presentations) the intra-subject agreement rate was 39%. While we expect that the intra-subject rate would be lower, and more directly comparable to the inter-subject rate, had the repeat presentations been made in different sessions on different days, there is clearly a substantial margin between the two rates. Hence our data should certainly be considered to show the variability of naming across a population, rather than within a single subject.

To clean the data common spelling variations (e.g. gray/grey, redish/reddish) were made consistent. Then responses that were unique to a single subject (e.g. *vague pink*, *easter green*) were eliminated. These unique responses made up ~10% of the data. Without repeats of a response by different subjects we have no confirmation that it is not individual and idiosyncratic, and it is difficult to draw any conclusions about the extent of the corresponding colour category. After elimination of unique responses, and of responses to the repeated presentation, 18,643 responses remained using 804 distinct terms. In the remainder we call these *names*.

## 4. Statistics of naming data

**Fig 4:**
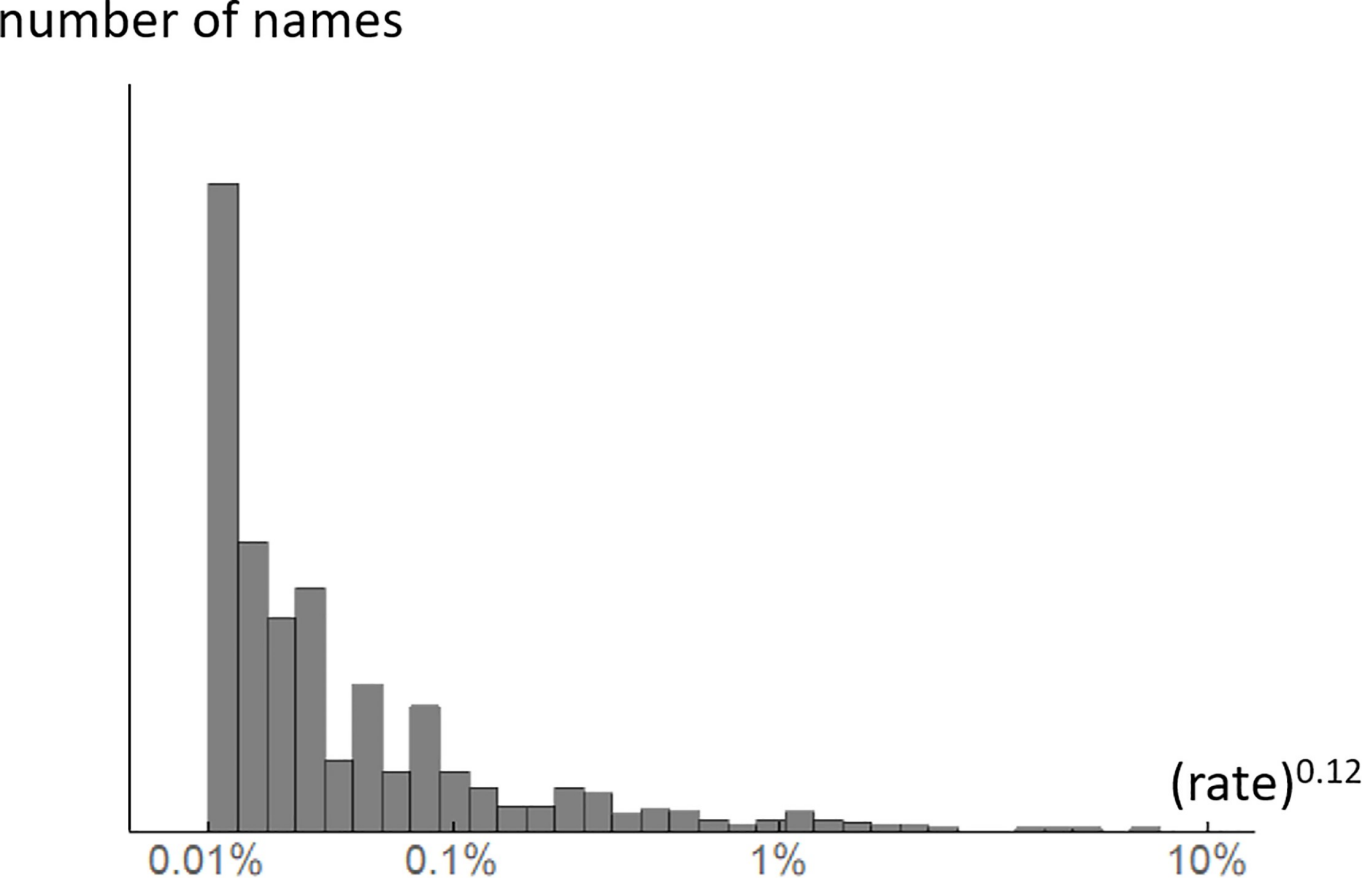
Histogram of global response rates for different colour names showing an exponential form. Note the non-linear horizontal axis.

The number of responses per name across all chips (global response rate) varies hugely. The most frequent response (*purple*) was recorded 1,346 times, hence a global response rate of 7.2%; while the least frequent (e.g. *bluey green*) were produced only twice, hence 0.01%. The distribution of global naming rates is extremely skewed, but we find that after power-law transform (exponent 0.12) their distribution has an exponential form (Fig 4). We will make use of this lawful distribution of response rates as prior when we fit a model to the response data in section 5.

**Fig 5:**
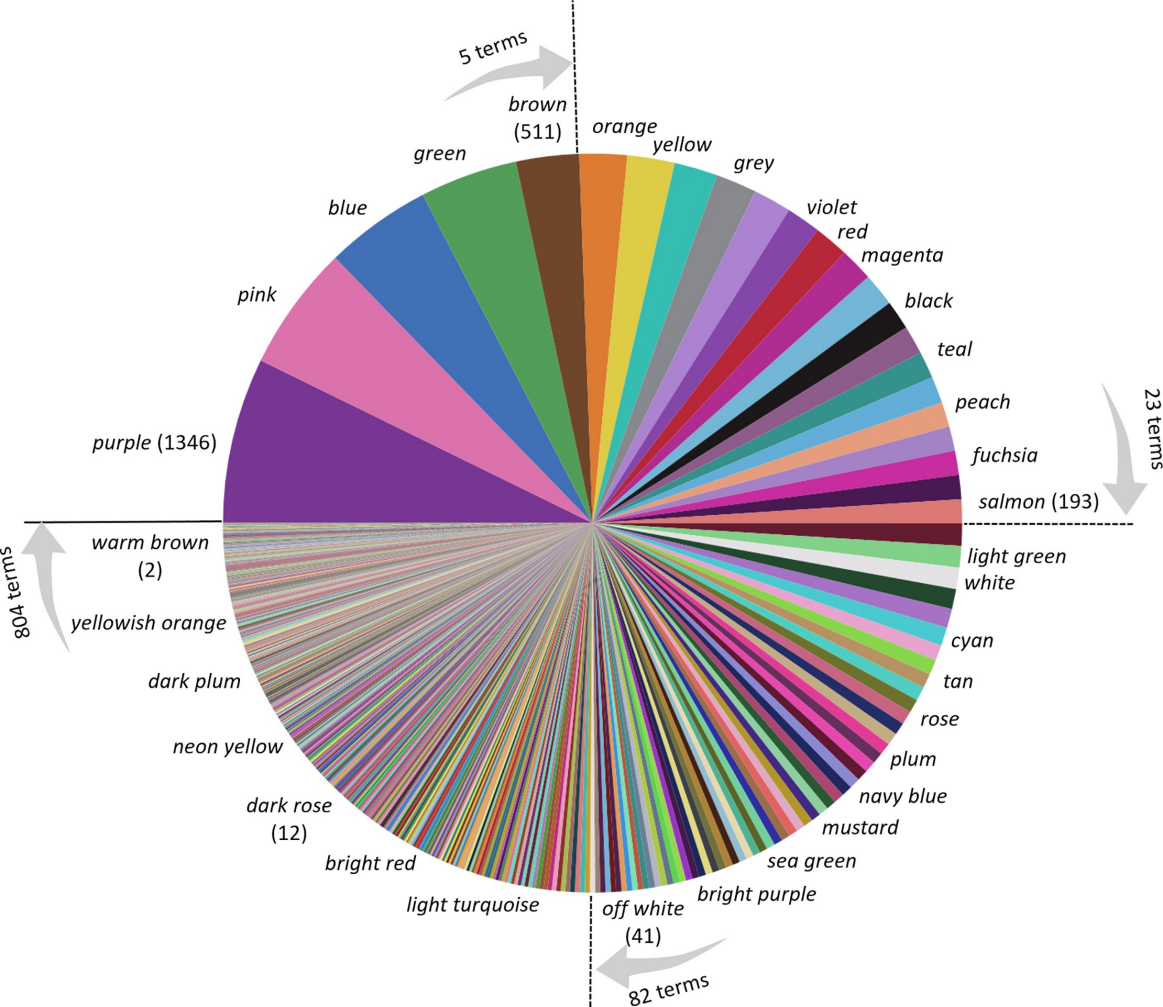
Breakdown of responses by name, ordered by their global frequency. Sectors are coloured by the mean colour of the chip generating the response. A subset of sectors are labelled by name, and a subset of those also show the number of responses in brackets.

The piechart in Fig 5 is a second way to visualize the distribution of names recorded for chips. It shows that the five most frequent responses (*purple*, *pink*, *blue*, *green* and *brown)* accounted for one quarter of all responses; the 23 most frequent names accounted for half, and included non-basics such as *violet* and *teal*; the most frequent 82 names accounted for three-quarters and included multi-word responses such as *light green*, *navy blue* and *off white*. 88% of responses were with names that were produced ten or more times.

Finally we consider the entropy of the distribution of responses – globally and per chip. The entropy of the global distribution of responses is 7.3 bits (i.e. the same as a uniform distribution across 154 names) less than the 9.7 bits had the distribution been uniform across the 804 names produced. The mututal information between chips and names is 4.2 bits – this is how much information is provided by the name evoked, on average, about which chip it was that caused the name. The mean entropy of the per chip response distributions is 3.1 bits; examples for individual chips are:

smallest: entropy = 0.6 bits; 〈0.00,0.00,0.00〉_*sRGB*_; 31×*black*, *very dark purple*, *dark brown*, *blue*.
median: entropy = 3.2 bits; 〈0.85,0.66,0.14〉_*sRGB*_; 10×*yellow*; 4×*orange*, 4×*mustard*, 4×*gold*, *2*×*yellow orange*, 2×*mustard yellow*, 2×*golden yellow*, 2×*goldenrod*, *tangerine*, *sunflower yellow*, *golden orange*, *bright orange*.
largest: entropy = 4.4 bits; 〈0.60,0.71,0.66〉_*sRGB*_; 3×*grey*, 2×*pale green*, 2×*greeny grey*, 2×*green*, 2×*aquamarine*, *turquoise*, *sea foam*, *sage*, *robins egg*, *pastel green*, *pale turquoise*, *pale grey blue*, *pale aqua*, *muddy green*, *mint green*, *mint*, *light blue green*, *light blue*, *ice blue*, *dark mint*, *baby blue*, *aqua blue*, *aqua*.

## 5. Modelling naming data

In this section we will show that computing the categorical metric directly from response data is problematic, and instead propose to derive the metric from a model fitted to the data. We will then describe how we fitted such a model and evaluate its quality.

To compute the categorical metric throughout the RGB cube we would, ideally, have access to the exact population distribution of naming responses for all possible colours. In practice we have access only to *sampled* distributions of responses, and at only certain locations (600≈8.4^3^ chips). Sample statistics can give biased estimates of population statistics. For some measures (e.g. variance of a distribution) an effective bias correction is known; while for others (e.g. Bhattacharya angle between two distributions) it is not. We mention the Bhattacharya angle as it is closely related to the computations needed to determine the categorical metric. We demonstrate the severity of its sample-based bias using a model example as follows.

In our collected naming data a typical chip has 31 responses distributed across a median 13 names with an entropy of 3.2 bits. We can model the response distribution for this typical chip as a multinomial distribution with probabilities that decay with rank (*r*) proportional to *r*^−1.4^; thus 34%, 13%, 7%, 5%, etc. Sets of 31 samples drawn from this model distribution have a median of 15 distinct responses and an entropy of 3.2, reasonably matching our typical chip (the exponent -1.4 was chosen to achieve this). Now consider two populations with this exact same model distribution. The Bhattacharya angle between them is zero (because they are identical), but the mean Bhattacharya angle between two independently-drawn 31-sample distributions is 0.85 (standard deviation 0.10). This is a severe over-estimate given that Bhattacharya angles are necessarily in the range [0,π2]≈[0,1.6].

Rather than trying to correct such biases we instead fit a parametric model to the dataset of responses across all chips, and from this model infer population distributions at any location. Robustly fitting a model to the response data is plausible since (i) we have many responses, (ii) at many well-spaced chips, (iii) we can make reasonable regularizing assumptions about the spatial structure of the response rate for each name; in particular that it will be roughly unimodal and will not change too fast, and (iv) in section 4 we observed a lawful distribution on global response rates which we can use as a prior for regularization.

**Fig 6:**
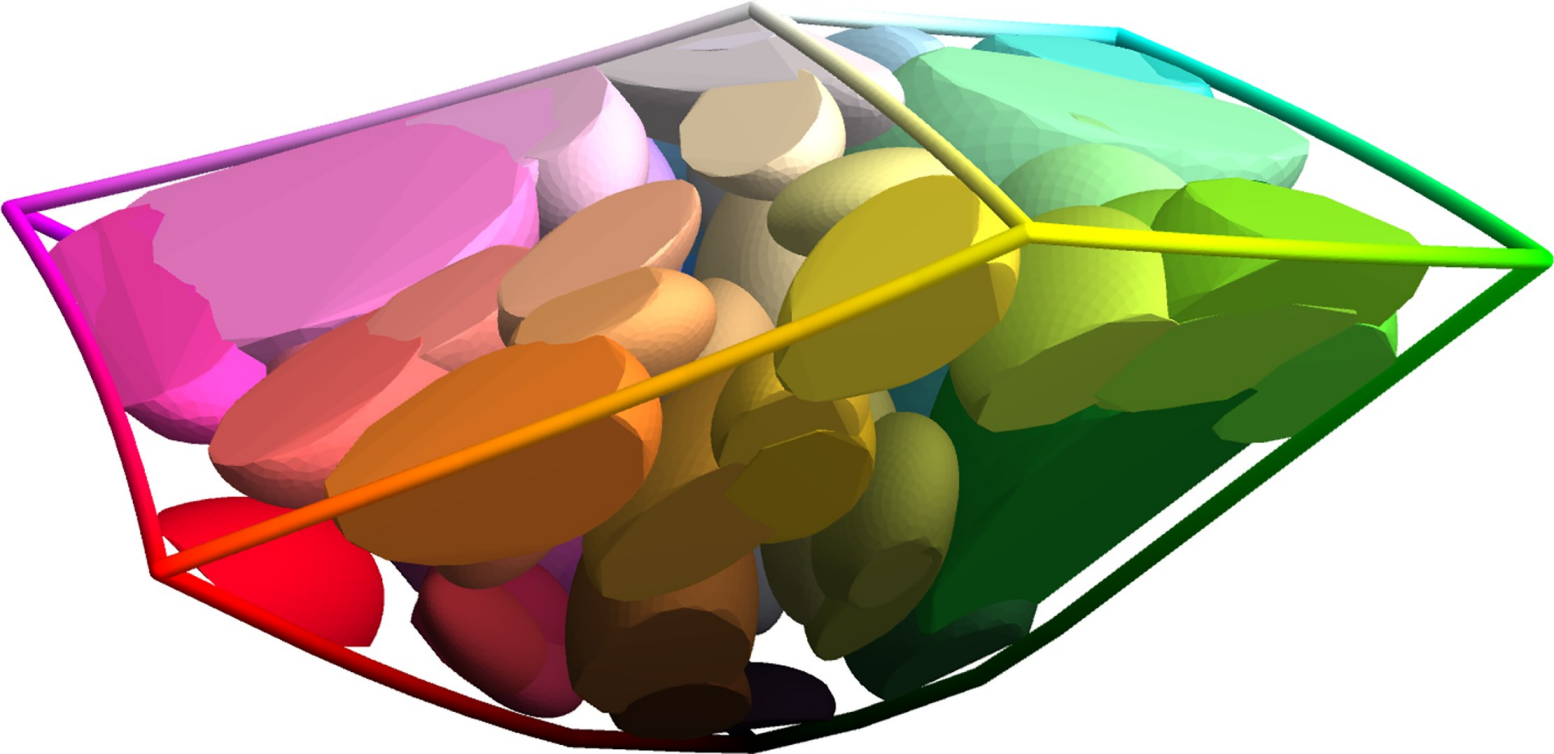
Visualizes, in CIELAB space, the individually-fitted response rate functions for the 82 most frequent names, which together account for 75% of all responses. Each response function has a unimodal form decaying from a peak response rate in the centre. The ellipsoids mark where the response rate has reduced to half the peak value. The figure does not visualize the peak response rate itself. The CIELAB distance from the magenta corner (left) to the green corner (right) is 225.0 units.

We model the response rate for name *n* at colour $\vec{z}$ as $Q_{n}\left(\vec{z}\right)≔\gamma g_{n}+\left(1-\gamma \right)k_{n}e^{-\frac{1}{2}{\left({\left(\vec{z}-{\vec{\mu }}_{n}\right)}^{T}\Sigma_{n}^{-1}\left(\vec{z}-{\vec{\mu }}_{n}\right)\right)}^{\alpha_{n}}}$; a refinement of a previous model [68]. This has a unimodal form (as per assumption) which peaks at rate at colour location ${\vec{\mu }}_{n}$, and decreases with distance from that location controlled by a 3×3 covariance matrix **Σ**_*n*_ and by a sharpness parameter *α*_*n*_. If *α*_*n*_ = 1 then the rate reduces with a Gaussian falloff, while with higher values of *α*_*n*_ the fall-off is initially slower but then more rapid. We ensure that the eigenvalues of **Σ**_*n*_ are greater than 3.0^2^ (CIELAB distance) to prevent categories being implausibly tight – see Fig 6 to visually gauge this. We model erratic responses by our subjects, which are independent of the stimulus viewed, using a small lapse rate *γ*≔0.01 times *g*_*n*_ the global response rate for *n*.

We fit the response function of each name independently. If the observed number of *n* responses at a chip $\vec{c}$ is $r_{n}\left(\vec{c}\right)$ and the number of non-*n* responses is $s_{n}\left(\vec{c}\right)$ then the log-likelihood of the data according to the model is $L_{n}≔\sum_{\vec{c}}\left(b_{n}\left(\vec{c}\right)+r_{n}\left(\vec{c}\right).\ln Q_{n}\left(\vec{c}\right)+s_{n}\left(\vec{c}\right).\ln\left(1-Q_{n}\left(\vec{c}\right)\right)\right)$, where $b_{n}\left(\vec{c}\right)=\ln\left(\begin{array}{c} r_{n}\left(\vec{c}\right)\\ r_{n}\left(\vec{c}\right)+s_{n}\left(\vec{c}\right) \end{array}\right)$ is a combinatoric term using the binomial coefficient function. We use conjugate gradient ascent to optimize $k_{n},{\vec{\mu }}_{n},\Sigma_{n},\alpha_{n}$ to maximize the log-likelihood minus a penalty term i.e. $L_{n}-\lambda .{\left(k_{n}\sqrt{\left|\Sigma_{n}\right|}\right)}^{0.12}$. The penalty term models a prior that the overall response rate for a name (across the RGB cube) should be exponentially distributed after power-law transformation (exponent 0.12) as shown in section 4. The effect of this prior is to encourage response functions with smaller support and lower peak. Without this prior, counter-intuitive maximum likelihood solutions can be found for names with small numbers of responses. For example their support may be long and thin, or they can peak away from the chips where the responses were recorded at a much higher rate than observed at any chip. We tuned *λ* using cross-validation (fit responses on a random half of the chips, test on the other half), finding that a value of *λ*≔100 was optimum. Examples of fitted response functions are shown in Fig 6.

To combine the response functions for individual names, in order to predict full distributions of responses, we normalize them to unit sum i.e. $P_{n}\left(\vec{z}\right)≔Q_{n}\left(\vec{z}\right)/\sum_{s}Q_{s}\left(\vec{z}\right)$. Jointly optimizing all response functions simultaneously, evaluating likelihood from the *P*_*n*_ rather than from individual *Q*_*n*_, should produce a better fit to the full naming dataset. However, not only is the computational challenge of this joint optimization considerable, but solutions obtained by it are prone to counter-intuitive aspects that are avoided with the per-name approach. It seems that the per-name fitting procedure has a regularizing effect.

**Fig 7:**
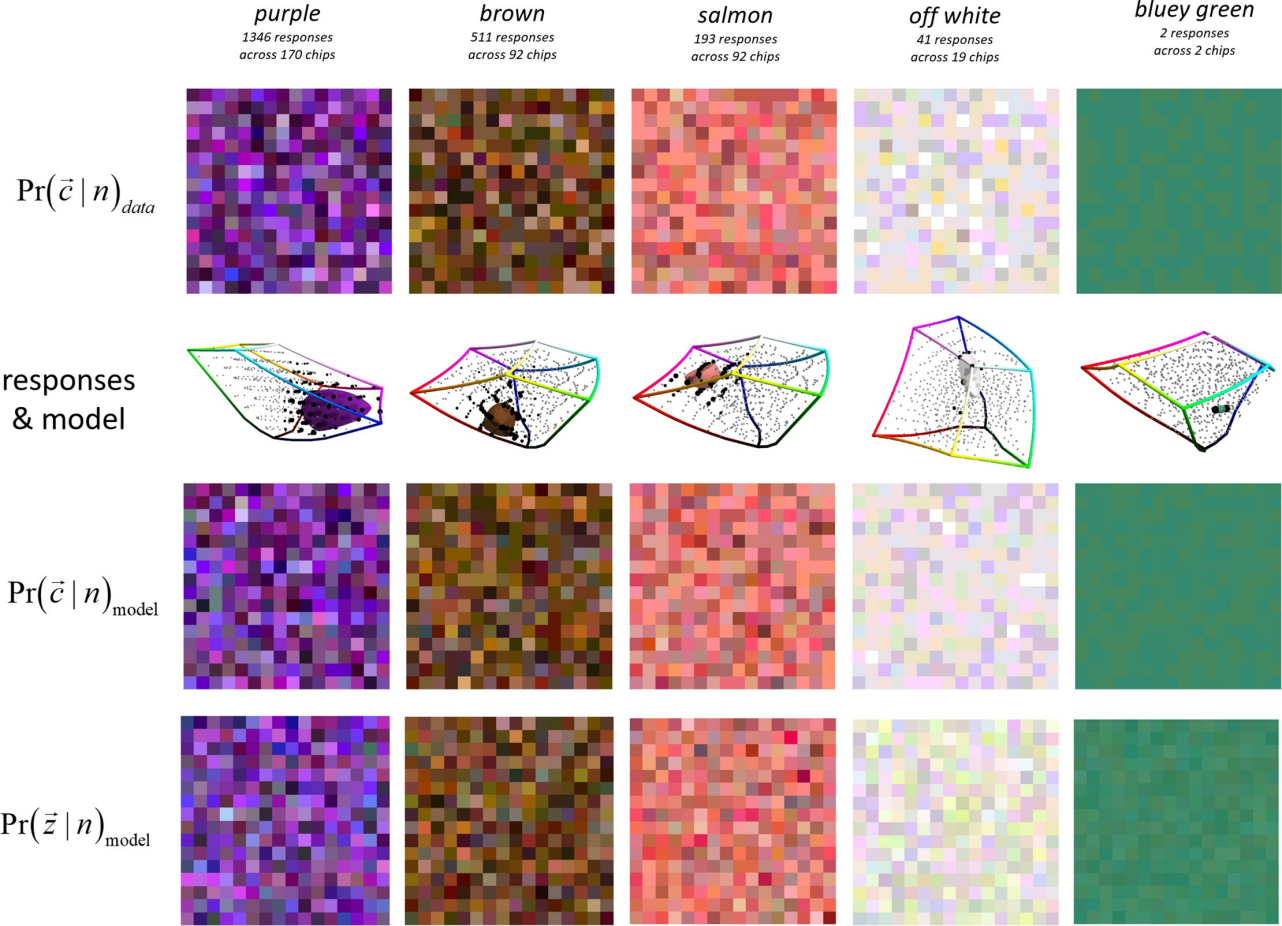
Each column shows the data and model for a different name *n* (shown in the top row). 2^nd^ row: samples from the distribution of chips causing naming response *n*. 3^rd^ row: the CIELAB locations of the chips causing response *n* are shown as black spheres, with volume proportional to the response fraction for that chip; other chips are shown as smaller grey spheres; the one sd limit of the fitted covariance is also shown. 4^th^ row: like the 2^nd^ row but according to the fitted model rather than direct from the data. 5^th^ row: generalization of the 4^th^ row from the 600 chips used as experimental stimulus to the full colour gamut.

Having established the *P*_*n*_ functions we perform sanity checks on the fitted model. In Fig 7 we compare, for a representative selection of names, colours picked according to ${\mathrm{Pr}}_{data}\left(\vec{c}|n\right)$ and ${\mathrm{Pr}}_{\mathrm{model}}\left(\vec{c}|n\right)$. These are the conditional distributions of which chip was the stimulus given the name produced as response. We have compared the data and model distributions for many more names than shown in the figure, and in all cases there is excellent visual correspondence.

Fig 7 also shows ${\mathrm{Pr}}_{\mathrm{model}}\left(\vec{z}|n\right)$ which is the conditional distribution of colours (not chips) being the cause of a name being produced (by a synthetic observer acting like the model), assuming a uniform prior in CIELAB across the extent of the RGB cube. These distributions are a generalization from the particular chips we used experimentally to the full colour manifold. In all cases that we have inspected the generalization seems good. The regularization term ($-\lambda .{\left(k_{n}\sqrt{\left|\Sigma_{n}\right|}\right)}^{0.12}$) in the fitting of response functions is critical to this consistent good generalization.

**Fig 8:**
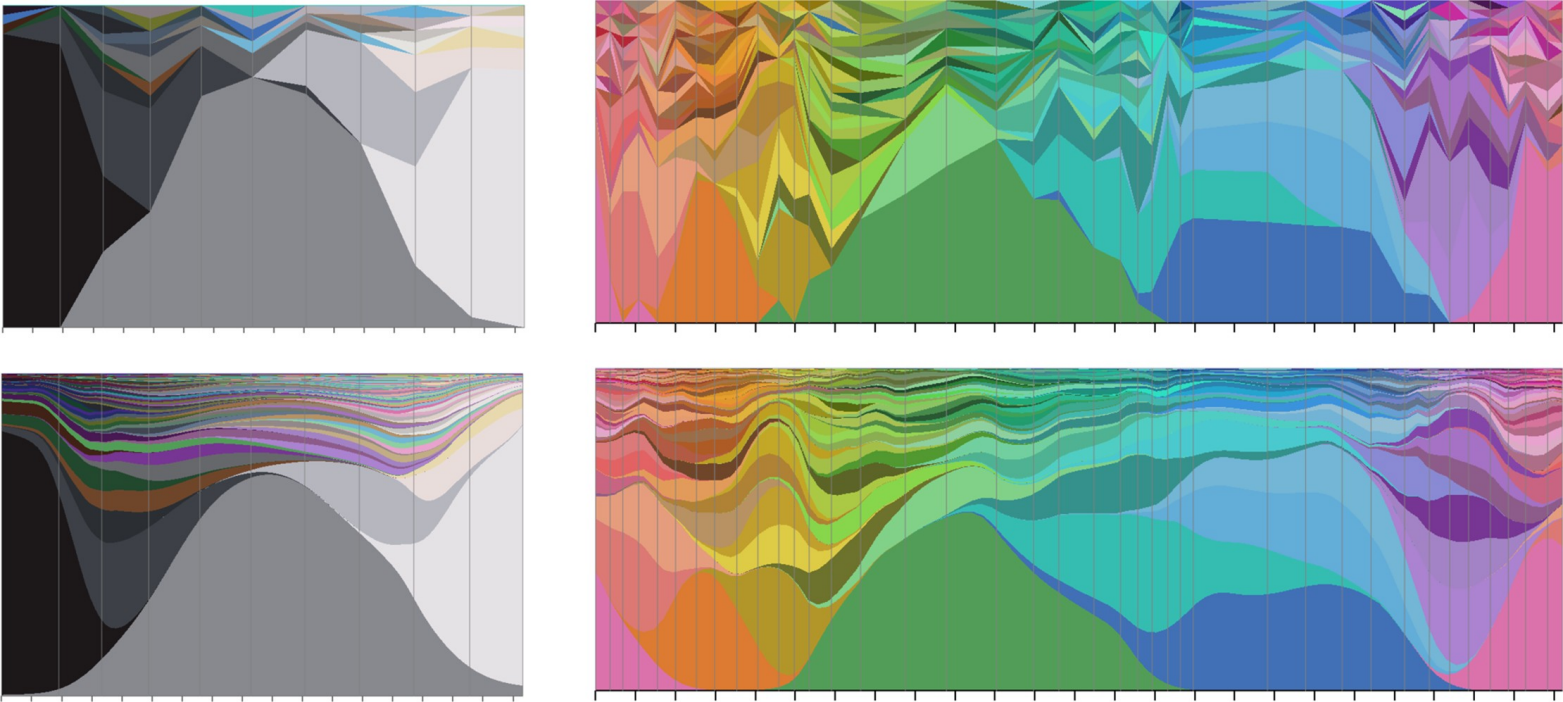
Left: Visualizations for a path along the achromatic axis. Right: for a circular path around the achromatic axis (see Fig 1 right). Top: Response distributions, piecewise linearly interpolated along sRGB paths, tied to observed response distributions at chip locations marked by grey vertical lines. Bottom: modelled response distributions along the same paths. In all panels the tick marks along the horizontal axis have spacing Δ = 0.1 (sRGB units), the smoothness length scale used in section 6. Vertical sections show proportions of naming responses (each colour corresponds to a different name) as in Figs 2 and 3.

Our next assessment of the model (see Fig 8) is to visualize the observed and modelled response distributions along continuous paths through colour space which pass through the locations of multiple chips. The figure shows that the model is capturing the trends of the observed response distributions well, while ironing out what can be attributed to sampling variability, resulting in response functions that are smoother and more unimodal than the observed data

To formally assess the quality of the fitted model we compare the likelihoods of the observed data relative to it, and relative to baseline and saturated models. The baseline model uses the same fixed response distribution for each chip, given by the global response rates also used for modelling lapses i.e. $g_{n}≔\sum_{\vec{c}}r_{n}\left(\vec{c}\right)/\sum_{s,\vec{c}}r_{s}\left(\vec{c}\right)$. The saturated model uses the observed rates for each chip i.e. $r_{n}\left(\vec{c}\right)/\sum_{s}r_{s}\left(\vec{c}\right)$. When comparing models through likelihoods, the rule of thumb (from comparison of AIC values [69]) is that deviances (twice the difference in likelihood of two models) should be compared to the difference in the number of model parameters to determine which of two models offers the better trade-off between model fidelity and complexity [70]. Applying this rule to Table 1 shows that our fitted model is preferable to the baseline model i.e. 2×(62,095−17,520) = 89,150 ≫ 8,917 = (9,720−803), and to the saturated model i.e. 2×(17,520−8,201) = 18,638 ≪ 472,080 = (481,800−9,720).

**Table 1:** Comparison of models.

model	ln likelihood	number of model parameters
baseline	-62,095	803 = *N*_*names*_−1
fitted	-17,520	9,720 = 12*N*_*names*_
saturated	-8,201	481,800 = *N*_*chips*_×(*N*_*names*_−1)

## 6. Computing metrical structure

The categorical metric tensor at a colour depends on derivatives (with respect to colour) of the square-rooted response distributions. In principle, given the fitted model of response distributions we could analytically compute the needed derivatives at any required colour location, and from those compute the tensor. This would be extremely cumbersome in practice. The more convenient strategy we pursue is to compute and store the tensor at a sampled grid of colours, and estimate the tensor at intermediate colours using interpolation as needed. We base the sampling of our scheme around the RGB length Δ≔0.1 (approximately 10.0 in CIELAB units) – this is roughly the confusion scale for colours [29] and so it is unlikely that categorical structure has non-artefactual articulations on shorter length scales than this.

To prepare the signal for sampling we apply a small blur to the fitted response distributions (before square rooting), and by careful choice of the step size used when computing finite-difference derivatives of the square-rooted blurred distributions. As established in the context of image processing, appropriately matched spatial blurring and finite differences together approximate computation of derivatives by inner product with derivative-of-Gaussian filters, which has been shown to be optimal [71].

For blurring the fitted response distributions we use a 3-D outer product of a 1-D five-point dirac-comb averaging filter with spacing Δ/3 and weights 112[13431], hence 1-D spatial spread (standard deviation) of 0.36Δ = 0.036. The Δ/3 step size of this filter is sufficiently small to prevent aliasing given the lower bound on the eigenvalues of the fitted response functions covariance matrices (**Σ**_*n*_), and the maximum permitted value (3.0) of the sharpness parameters (*α*_*n*_). Also consider the smoothness visible in Fig 8 which has Δ-spaced tick marks.

After blurring, when computing finite differences we use a step size of Δ. The roughly 3:1 ratio between the blur width and step size results in a good approximation to a derivative of Gaussian filter. We denote the blurred, fitted response distribution as $M\left(\vec{c}\right)$.

We compute derivatives, at the colour $\vec{z}$, of the square root of *M* in the R-, G- and B-directions e.g. $dR≔\Delta^{-1}.\left(\sqrt{M\left(\vec{z}+{\left(\Delta /2\;0\;0\right)}^{T}\right)}-\sqrt{M\left(\vec{z}-{\left(\Delta /2\;0\;0\right)}^{T}\right)}\right)$. Each derivative is a 804-D vector, the same as the distributions. The metric tensor is then computed as **Γ**≔(*dR dG dB*)^*T*^(*dR dG dB*), yielding a 3×3 matrix.

**Fig 9:**
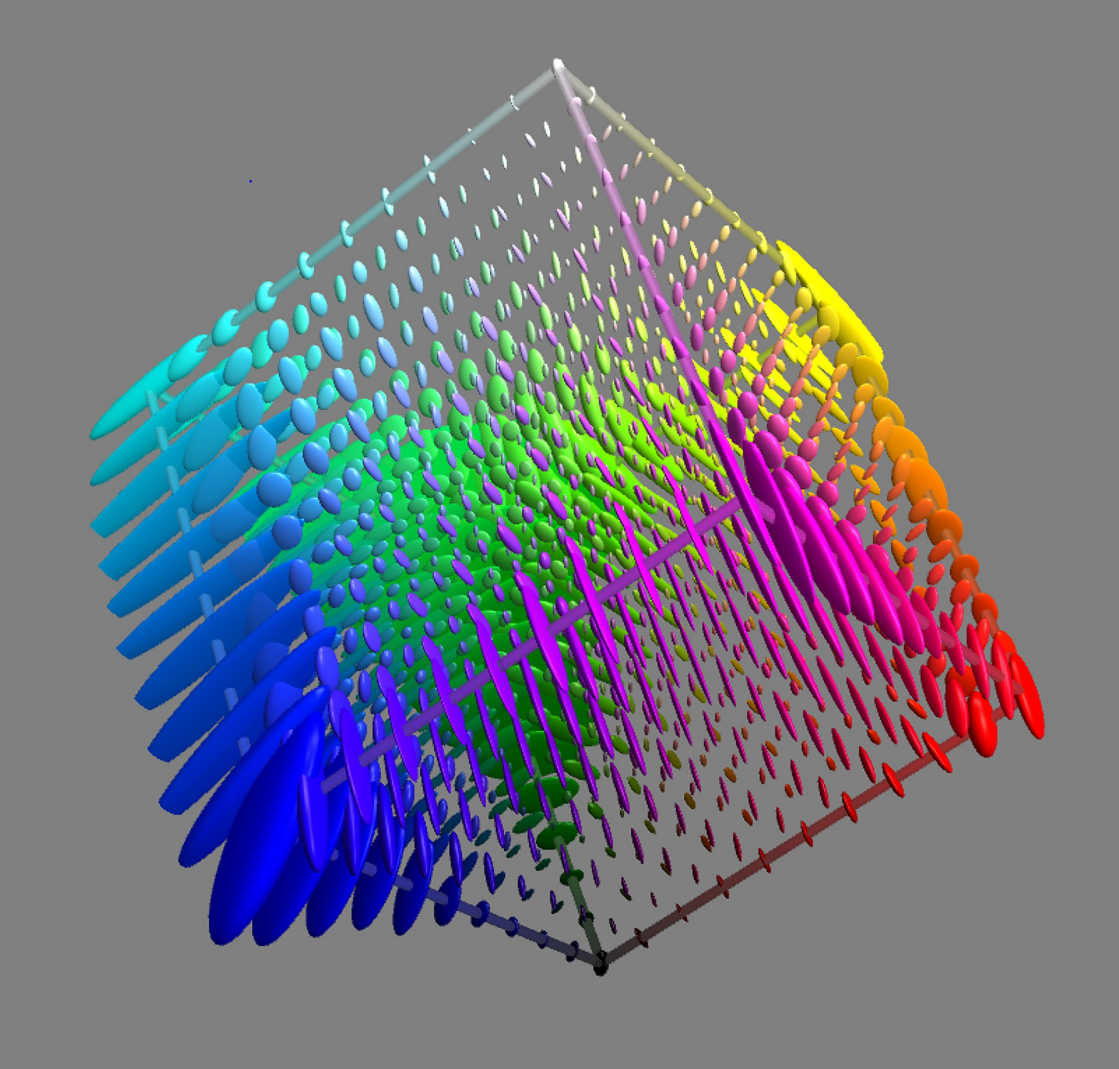
Tensor field of the categorical metric, visualized across sRGB space using a 3D version of Tissot’s indicatrix [72]. Each ellipsoid is a sphere from the perspective of the categorical metric, all spheres being of the same radius. That radius, chosen with an eye to visibility of the final figure, is in this case 1.6% of a grain. Where the ellipsoids are wide, categorical distance increases slowly with sRGB distance. So, for example, the large ellipsoid at the blue corner is indicating that there is very little difference between the naming response to colour 〈0.00,0.00,1.00〉_*sRGB*_ and to colour 〈0.05,0.05,1.00〉_*sRGB*_. On the other hand, where the ellipsoids are narrow, categorical distance increases more rapidly with sRGB distance. Hence, the small ellipsoid midway along the cube edge from cyan to white is indicating that the naming responses to 〈0.50,1.00,1.00〉_*sRGB*_ and 〈0.55,1.00,1.00〉_*sRGB*_ are noticeably different.

Our software implementation (in Mathematica) is able to compute the metric tensor at any RGB point in ~4s. This is inconveniently slow for some purposes. For faster computation we use quadratic interpolation of the tensor field pre-computed on a 3D grid of locations with spacing Δ/2. Since the metric tensor, by design, varies smoothly over a length scale of Δ this interpolation approach is accurate, as we have confirmed by numerical assessment. We have released the sampled grid of tensors as a supplement to this publication (https://doi.org/10.5281/zenodo.2595963). We visualize them in Fig 9.

## 7. Categorical geometry

The categorical metric, being Riemannian, has the potential to define a simple or a rich intrinsic geometry. The simplest it could turn out to be would be flat (Euclidean) in which case its geometry would be understandable as a simple warp into Euclidean space (as for example in CIELab). Of intermediate complexity it could have constant but non-flat curvature (like the surface of the earth). The most challenging would be for it to have varying non-constant intrinsic curvature, like the surface of a rugby ball, or worse. To add to the difficulty, the space is 3D not 2D making visualization and analogies difficult. We approach the problem by taking a tour through the categorical metric – holding it up to the light, so to speak, in different poses to develop an understanding.

We start (section 7.1) by assessing how much it differs from familiar metrics. Having established that it *does* differ substantially we attempt to gain familiarity with it first by examining it as a distortion of sRGB space (section 7.2), then by looking at its intrinsic shape by isometric embedding into Euclidean space (section 7.3). Lastly we will look at it in terms of the sizes of familiar parts (section 7.4).

### 7.1 Metrics compared

We wish to compare the categorical metric with more familiar colour metrics – to establish whether it is different, and how. When comparing two metrics (defined by their tensor fields **A**,**B**) we are interested in the different shape they define for the space, but not in overall changes of size. To achieve this we define *local distortion* as the log (base 2) of the ratio of the distances the two metrics assign to an infinitesimally close pair of colours $\vec{z}\pm \varepsilon \vec{v}$ i.e. $\frac{1}{2}\log_{2}\frac{{\vec{v}}^{T}A_{\vec{z}}\vec{v}}{{\vec{v}}^{T}B_{\vec{z}}\vec{v}}$. We then define *global distortion* as the standard deviation of local distortion. Assuming they are normally-distributed, the 95% range of local distortions can be estimated from the global distortion e.g. if the global distortion is 0.50 then local distortion will be in the range 2^±0.50×1.96^ = 51−197% for all but 5% of small colour distances.

To compute global distortion we integrate local distortion uniformly across all infinitesimally close pairs of colours. To ensure that this gives a symmetric comparison of the two metrics we use their geometric mean (C≔(AB−1)12B2(B−1A)12) to define the measure for the integration. We use a monte carlo approach (10^5^ samples) to integrate local distortion relative to **C**. Specifically we sample a $\vec{z}$ uniform randomly from the RGB cube; pick a colour direction as a random variate from the trinormal distribution $\vec{v}∼N\left(\vec{0},C_{\vec{z}}^{-1}\right)$; and then compute the local distortion (as previously described) with a sample weighting $\sqrt{\left|C_{\vec{z}}\right|}$. When we compute the global distortion ratio, as the standard deviation of the local, we take account of the sample weightings.

We have computed global distortion pairwise for a clutch of established distance measures in addition to the new categorical metric. Specifically, Euclidean distance in sRGB; Euclidean distance in CIELAB (also known as CIE1976); its refinement CIE1994; and the further refinement CIE2000.

To compute global distortion we requires distance measures to be in metric tensor form. We have already described how we computed the metric tensor for the categorical metric, and for sRGB the metric tensor is just the identity matrix. For the CIE distance measures we construct metric tensors as follows. At any particular colour location we first compute distances, according to the relevant CIE distance formulae, for 13 colour pairs Δ/2 apart (sRGB units), either side of the location. The 13 pairs are aligned along the axes and diagonals of the RGB system. From the 13 distances and separation vectors we estimate a compatible metric tensor by squared-error minimization.

**Fig 10:**
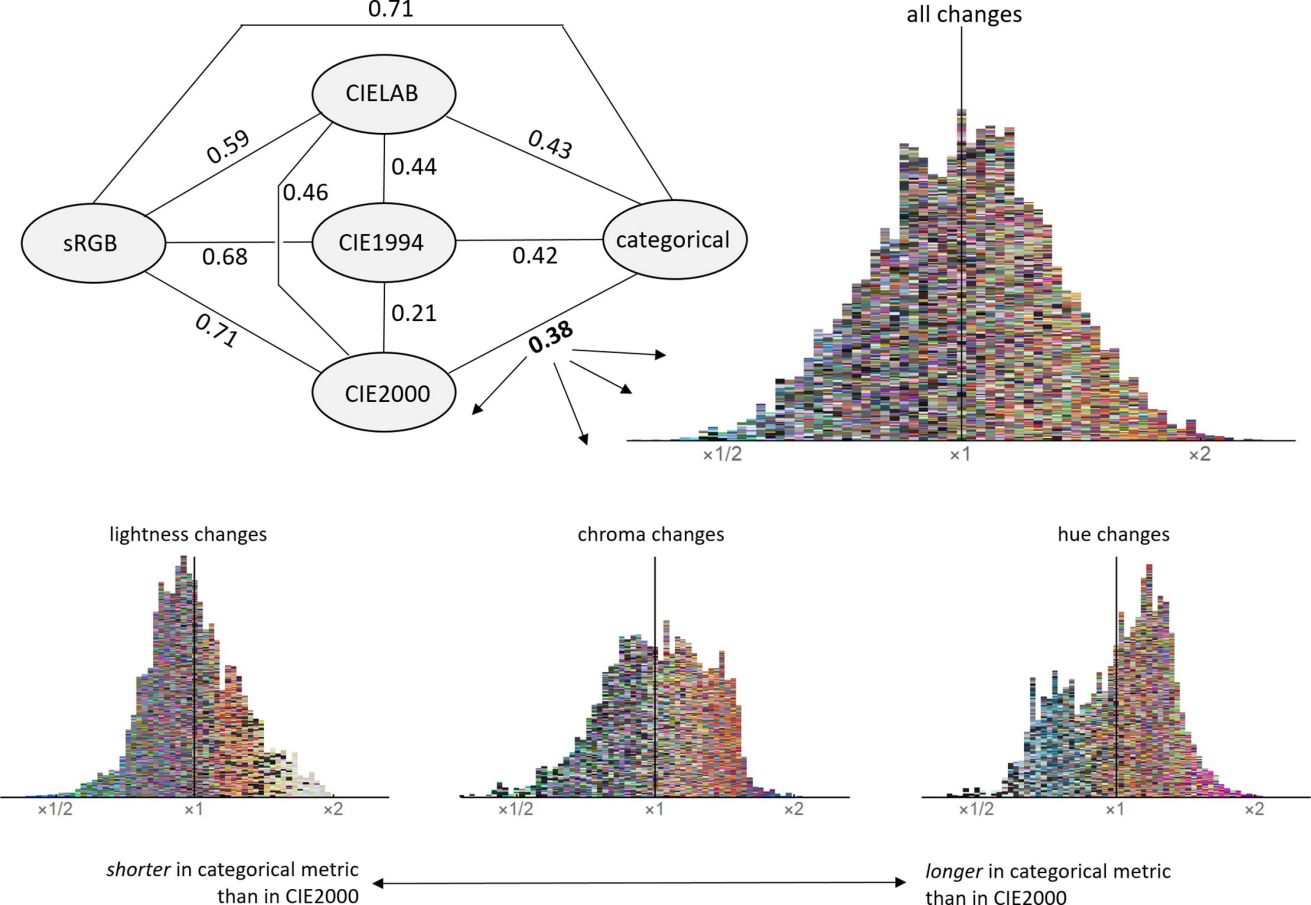
Distortion between metrics. Top-left: distortions quantified as the standard deviation of the log2 of the ratio of distances. Histograms: local distortions between CIE2000 and the categorical metric. Colours indicate the location of the colour difference (which is always small). The double arrow at the bottom of the figure applies to all four histograms.

The distortion measures are listed in Fig 10. They pass two sanity checks:

- Refinements of the CIE system are progressively more distorted compared to sRGB.
- The first refinement (CIELAB to CIE1994) was a larger change than the second (CIE1994 to CIE2000).

Comparing the categorical metric to other metrics:

- The categorical metric and CIE2000 are equally different from sRGB.
- Refinements of the CIE system are progressively more similar to the categorical metric.
- The distortion between CIE2000 and the categorical metric is larger than that between CIE1994 and CIE2000, and smaller than that between CIELAB and CIE1994.
- 95% of the local distortions between CIE2000 and the categorical metric are in the range 60-168%.

In Fig 10 we also visualize the distribution of local distortions between the categorical metric and CIE2000. The pattern is complex but some trends are visible:

- Distortions are equally manifest in lightness, hue and chroma changes.
- In warmer/lighter areas of colour space categorical distances tend to be greater than in CIE2000; and in cooler/darker, shorter. This is in line with the results of [73] which we interpret as showing that the extent (in JNDs) of colour categories in warm colour regions tend to be smaller than in cool.

The figure establishes that the categorical metric is not a simple adjustment of perceptual distance (as approximated by CIE2000).

### 7.2 Cross-sections

Given that the categorical metric is not a simple transformation of an already well-known metric we need to look at its detailed structure. Since the full 3D structure (Fig 9) is not easily interpreted, we try with 2-D cross-sections instead. To restrict a metric tensor **Γ** to a plane spanned by unit vectors $\vec{x},\vec{y}$ we form the 2D tensor ${\left(\vec{x}\;\vec{y}\right)}^{T}\Gamma \left(\vec{x}\;\vec{y}\right)$.

**Fig 11:**
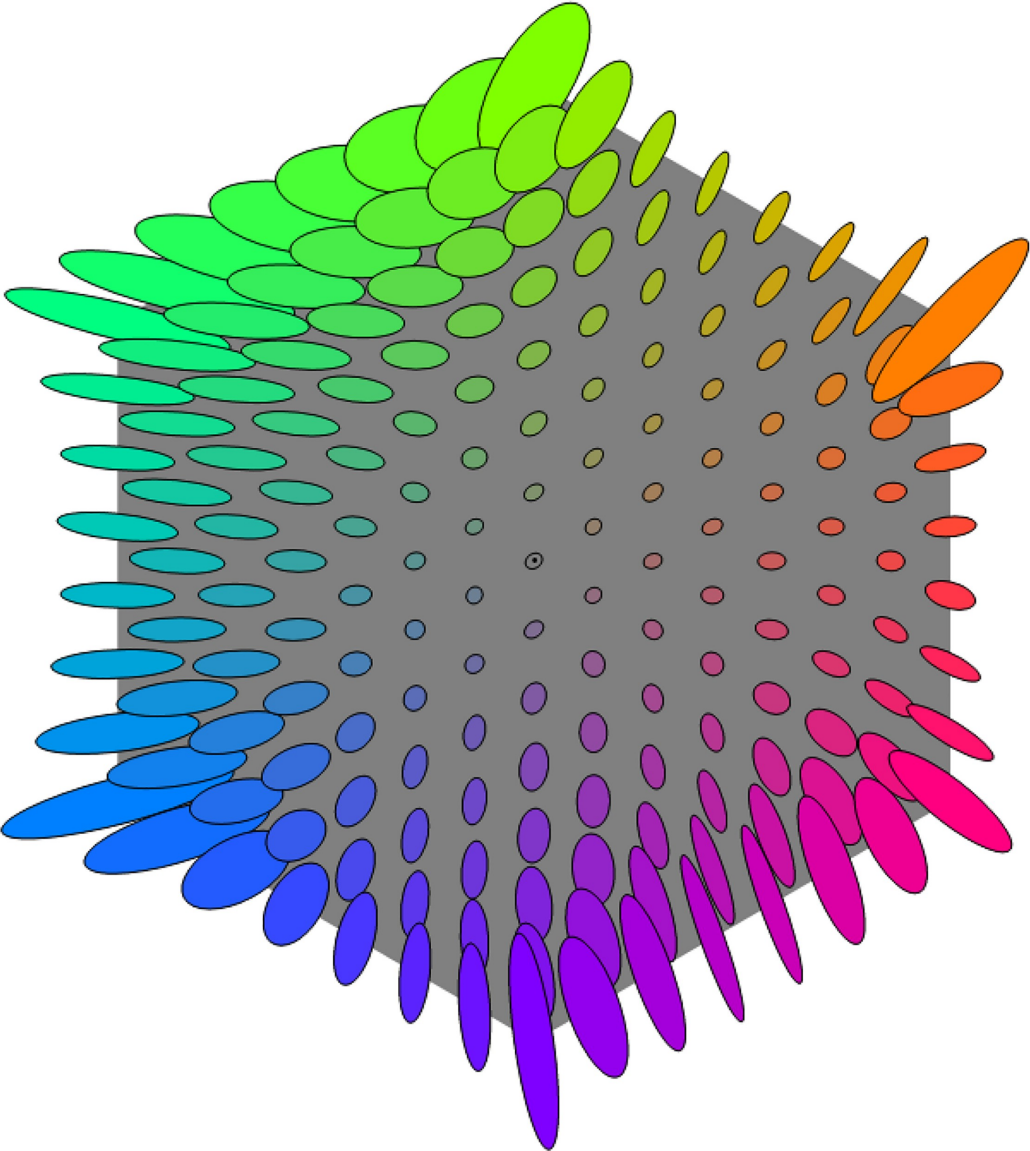
The categorical metric restricted to the mid-lightness section of the sRGB cube. See Fig 9 for interpretation of the ellipses. The corresponding sphere radius in this case is 5.3% of a grain.

In Fig 11 we show the categorical metric *within* the mid-lightness section of the sRGB cube. Fig 1 shows why this section is hexagonal. Where the ellipses are wide (hence small categorical change for an sRGB step) we describe the metric as slow; where narrow, fast – but note that these descriptors are not intrinsic, they are relative to the sRGB metric of the sectioning plane. Observations on the metric within the mid-lightness section are. 1. It tends to be faster at low saturation than high. 2. It tends to be faster for saturation than for hue changes. 3. Moving hue-wise around the more saturated colours, areas of slow metric (green, blue, purple, red, orange) alternate with areas of fast (yellow, green/blue, blue/purple, red/purple, red/orange). 4. The figure is directly comparable to Fig 18 in [29] for the colour matching geometry; that the two geometries are substantially different is immediately clear.

**Fig 12:**
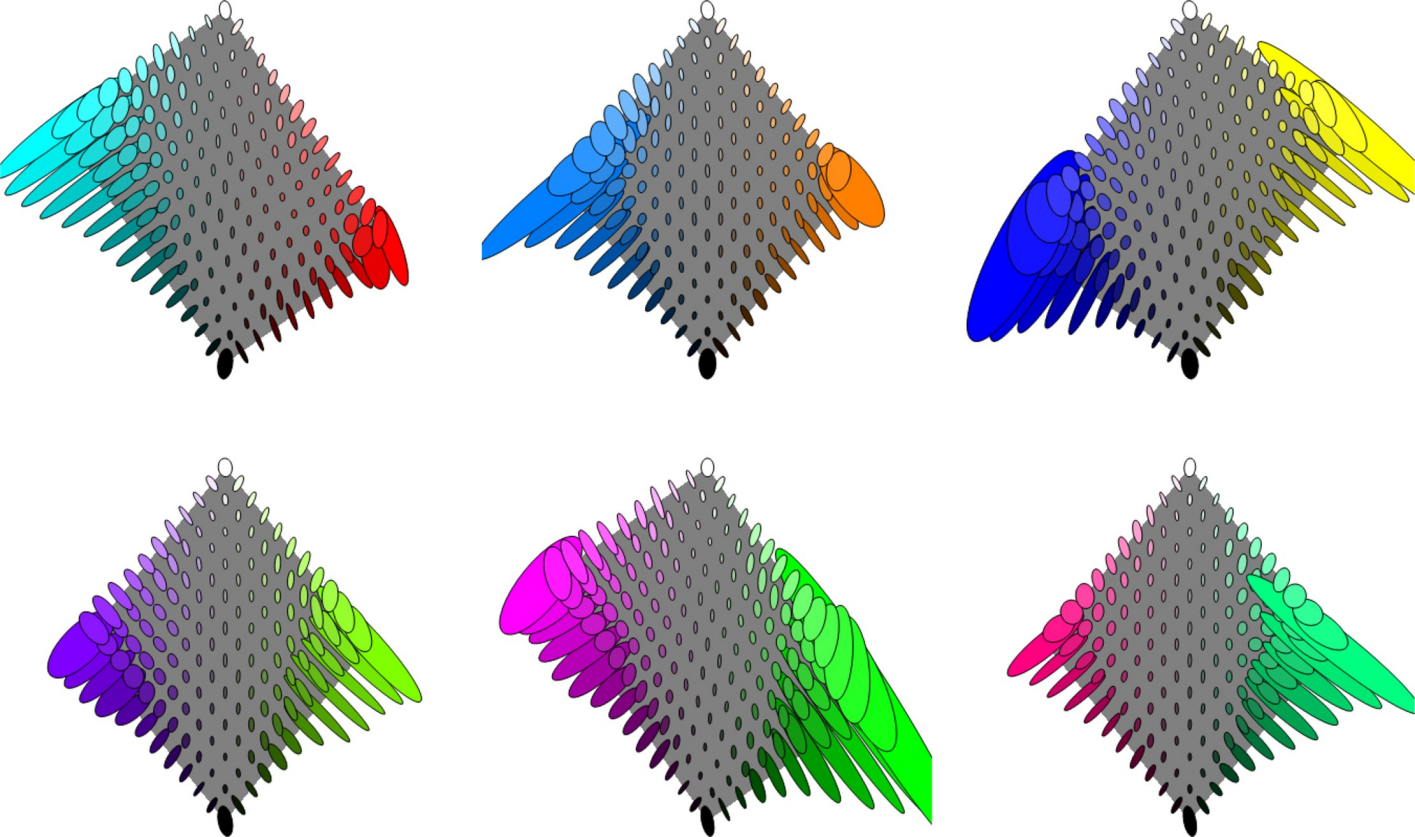
The categorical metric restricted to constant hue sections of the sRGB cube, with the achromatic axis running down the centre of each section. See Fig 9 for interpretation of the ellipses. The corresponding sphere radius in this case is 4.0% of a grain.

In Fig 12 we show the categorical metric within hue sections of the sRGB cube (see Fig 1). Some observations. 1. Consistent with the mid-lightness section, the metric has a clear tendency to be faster around the achromatic axis. 2. The variation with location of ellipse orientation is similar for all hues. 3. The metric along the achromatic axis is slowest at the ends, medium speed in the middle and fastest between the ends and middle. 4. Very large ellipses occur for most highly saturated colours, corresponding to there being very little change in naming distribution in those neighbourhoods. 5. The figure is directly comparable to Fig 17 and 23 in [29] for the colour matching geometry; that the two geometries are substantially different is immediately clear.

### 7.3 Embeddings

In the previous section we visualized the categorical metric as a distortion of sRGB. A different approach is to attempt to embed the manifold into Euclidean space, so that Euclidean distances match categorical distances [74–76]. Since the categorical metric may give the colour manifold non-Euclidean intrinsic curvature this is only guaranteed possible if we embed in a space of high enough dimension [77]. Specifically, if we seek an embedding free of self-intersections, then 2D is required for a 1D manifold, 4D for a 2D manifold, and 6D for a 3D manifold. If self-intersections are permitted, then a dimension one lower is sufficient.

**Fig 13:**
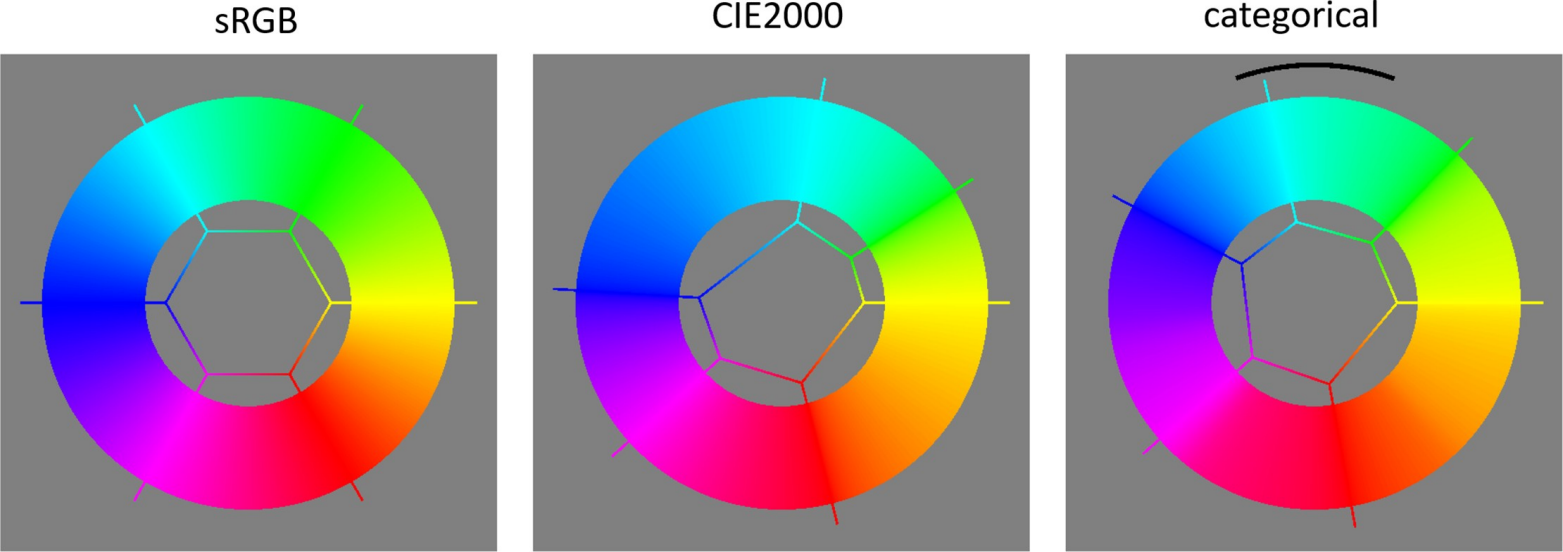
Each thick ring is an isometric embedding of the full colour locus (see Fig 1) according to the metric shown above the panel. The hexagons and spokes show the correspondence to the vertices and edges of the RGB cube along which the locus runs, and emphasize the differences between the embeddings. The arc in the right panel shows a one grain extent (see section 7.4).

In Fig 13 we embed the 1-D full colour locus sub-manifold (see Fig 1) into 2-D Euclidean space. For extra clarity we embed it as a circle. This is always achievable with zero distortion (as with the toy examples in section 2.1, the embedding is found by integration of the categorical metric). The panels contrast embeddings according to the sRGB, the CIE2000 and the categorical metrics. The hexagons in the figure mark the ‘corners’ of the full colour locus, and make it easier to compare the embeddings. Observations are. 1. There are clear differences between all three embeddings. 2. From CIE2000 to categorical the greatest changes are the shrinking of blue-cyan, and the expansion of blue-purple, followed by the expansion of yellow-green. 3. To the authors the colour banding is more uniform in the categorical embedding, but still not perfectly so. 4. The figure is directly comparable to Fig 11 in [29], and differences are clear.

**Fig 14:**
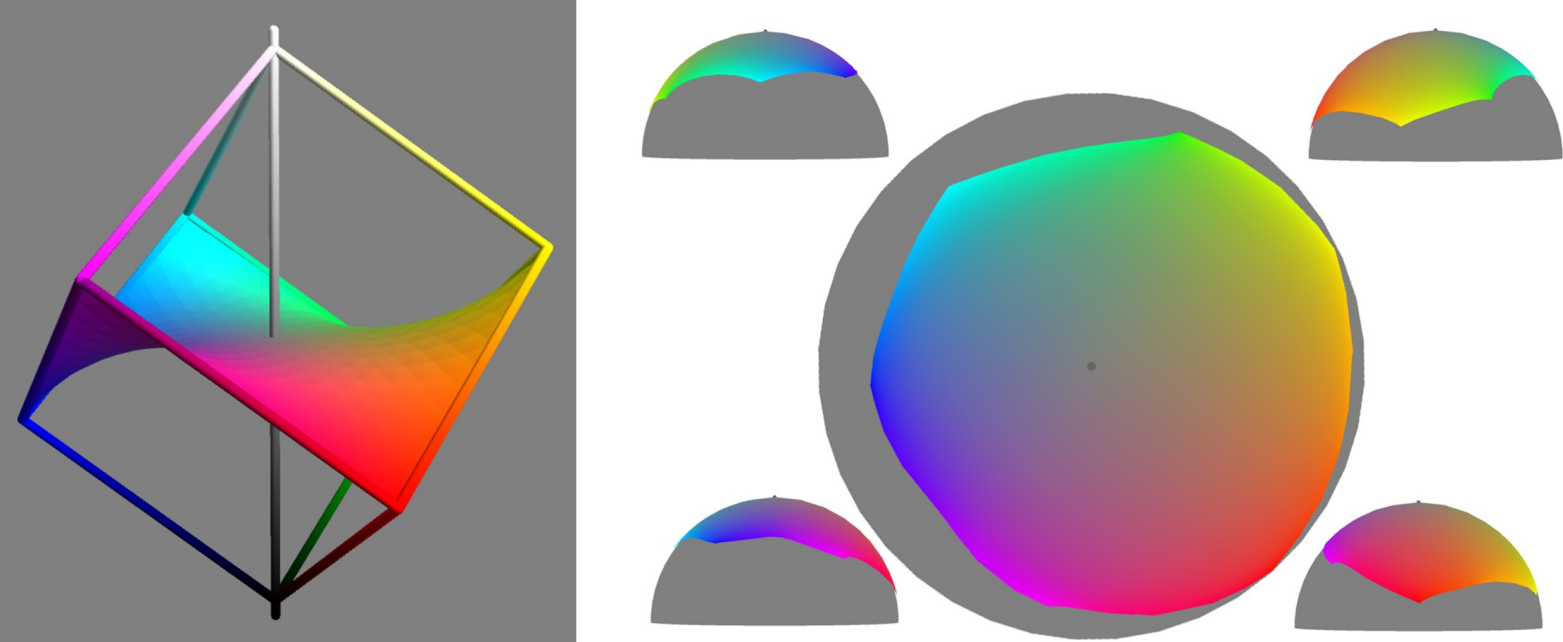
The full colour surface (left) is constructed by interpolation from the full colour locus (Fig 1). In sRGB it has a highly negatively curved form. Right: three views of a close to isometric embedding of the full colour surface onto a sphere.

Next we attempted an embedding of a 2D sub-manifold, the full colour surface. We construct the full colour surface by solving Laplace’s equation with the full colour locus as boundary condition. As can be seen in Fig 14 (left) it has a monkey saddle form, hence significant negative curvature (in sRGB). As a 2D manifold it can certainly be isometrically embedded into 3D, but since it turns out to have nearly constant positive Gaussian curvature, the embedding can be restricted to the surface of a sphere. Using numerical optimization of parameters controlling the sphere radius and the placement of mesh points onto the sphere, to minimize global distortion, this was achieved with 95% of local distortions in the range 92-109% - confirming the near constant curvature. Notably, a recent model of colour discrimination as determined by receptor noise also derived a geometry of constant positive curvature for the 2-D manifold of equiluminous colours [75]. Views of the embedding are shown in Fig 14 (right). Observations on the embedding. 1. Area of low saturation greatly expanded. 2. Areas around the six very saturated ‘tips’ are greatly reduced. 3. Overall shape is close to a spherical cap, with grey at the North Pole and the full colour locus in the range 12-43° latitude. 4. The expansion and compression of hues follows the pattern for the full colour locus (Fig 13). 5. Given that the full colour surface sits symmetrically within the RGB cube, its far from flat intrinsic curvature makes it unlikely that the full 3-D geometry is Euclidean (i.e. constant zero intrinsic curvature). Consequently we have not attempted an isometric embedding of the RGB cube into Euclidean space.

**Fig 15:**
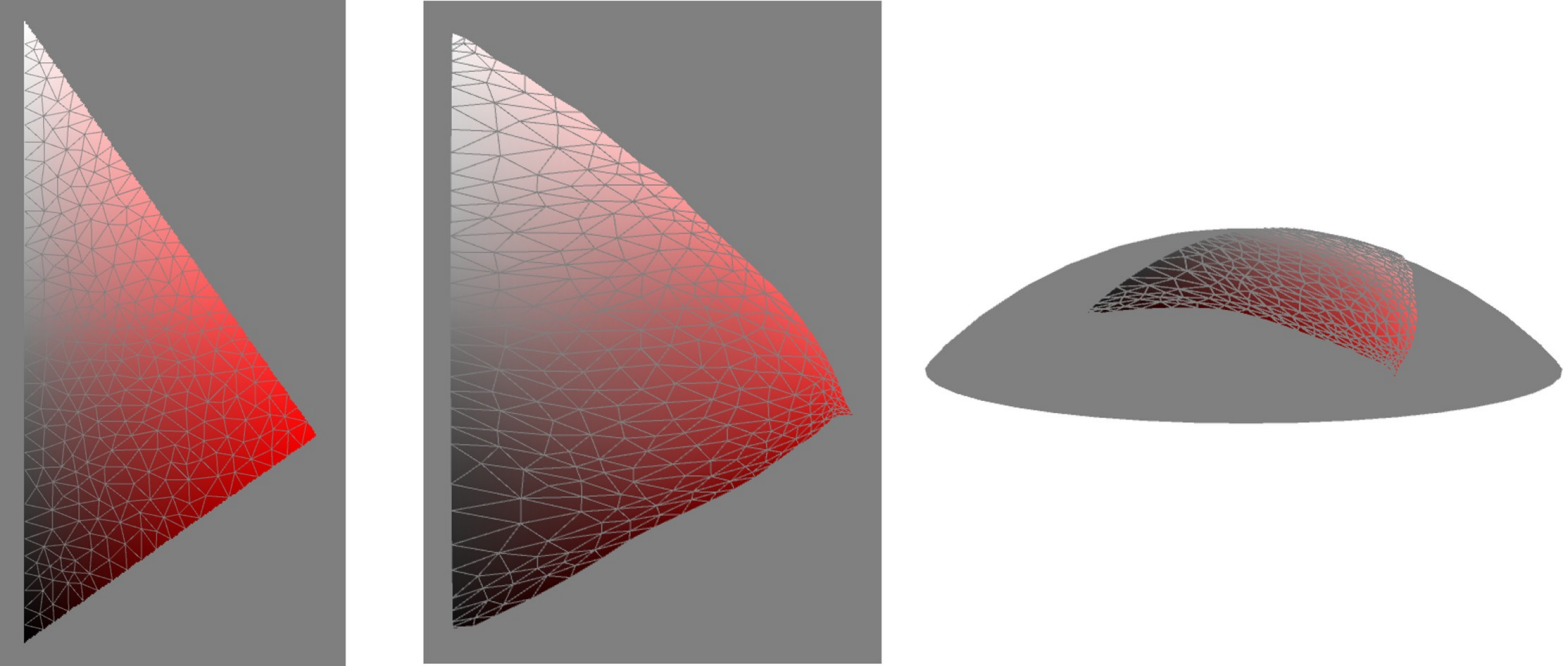
The red hue triangle from the sRGB cube (left). Embedded onto the surface of a sphere (middle and right) with only minor distortion.

Finally we embedded the red hue triangle from the sRGB cube (Fig 1). Again we found that the manifold had a close to constant positive Gaussian curvature, but closer to flat than the full colour surface. Embedding onto a sphere was possible with 95% of local distortions in the range 92-109% (see Fig 15). Other hue triangles (not shown) were similar, while varying in size (blue, for example, smaller). Observations. 1. Expansion of the low saturation territory compared to sRGB. 2. The red/white and red/black distances are more equal in the embedding than in sRGB, and the overall shape is close to equilateral.

### 7.4 Counting grains

In section 2.1 we introduced a *linear-grain* as a natural unit of categorical distance. We defined it as the distance between two distributions without possible responses in common, so π2 in raw categorical metric. We can use this unit of distance to define natural units of categorical 1-D extent, 2-D area and 3-D volume by defining them as the enclosure of a 0-, 1- and 2-sphere respectively of radius π4. Hence *G*_1_≔2(*π*/4) = *π*/2, *G*_2_≔*π*(*π*/4)^2^ = *π*^3^/16 and *G*_3_≔4*π*(*π*/4)^3^/3 = *π*^4^/48. We will refer to all these ‘sizes’ as grains, letting context disambiguate the dimension.

Measuring manifolds in terms of grains allows us to estimate how many categorically distinct regions of colour a manifold contains, which we call the categorical *capacity C*. As discussed for the toy models in section 2.1, it is important to consider the effect of the manifold boundary on capacity. There we showed that for a 1-D manifold of length *L* the capacity is given by $C≔L/G_{1}+\frac{1}{2}N_{endpoints}$. Observe how the additive correction is only significant when the number of grains is small, but note that this *is* the situation in colour so this is important. Back of the envelope arguments suggest that a general formula for the capacity of a manifold *R* of dimension *n* is $C\left(R\right)≔\left|R\right|/G_{n}+\frac{1}{2}\left|\partial R\right|/G_{n-1}$, where ∂*R* is the boundary of the manifold. This formula assumes that the curvature of the boundary is consistently low, if not it will under-estimate the capacity.

We start with 1-D sub-manifolds. The lengths, in grains, of the achromatic axis and full colour locus are 2.6 and 9.4 respectively. Their ratio of 3.6, compares to a ratio of 3.5 for the RGB cube and 2.8 for CIE2000. As the achromatic axis has endpoints, its capacity is $3.6=2.6+\frac{1}{2}\times 2$; as the full colour locus does not, its capacity is 9.4. These counts correspond reasonably with introspection: for achromatic, black, grey, white and a bit more for light and dark grey; for the full colour locus, six pure spectral hues – red, orange, yellow, green, blue and purple – plus three more, say cyan, magenta and chartreuse.

**Fig 16:**
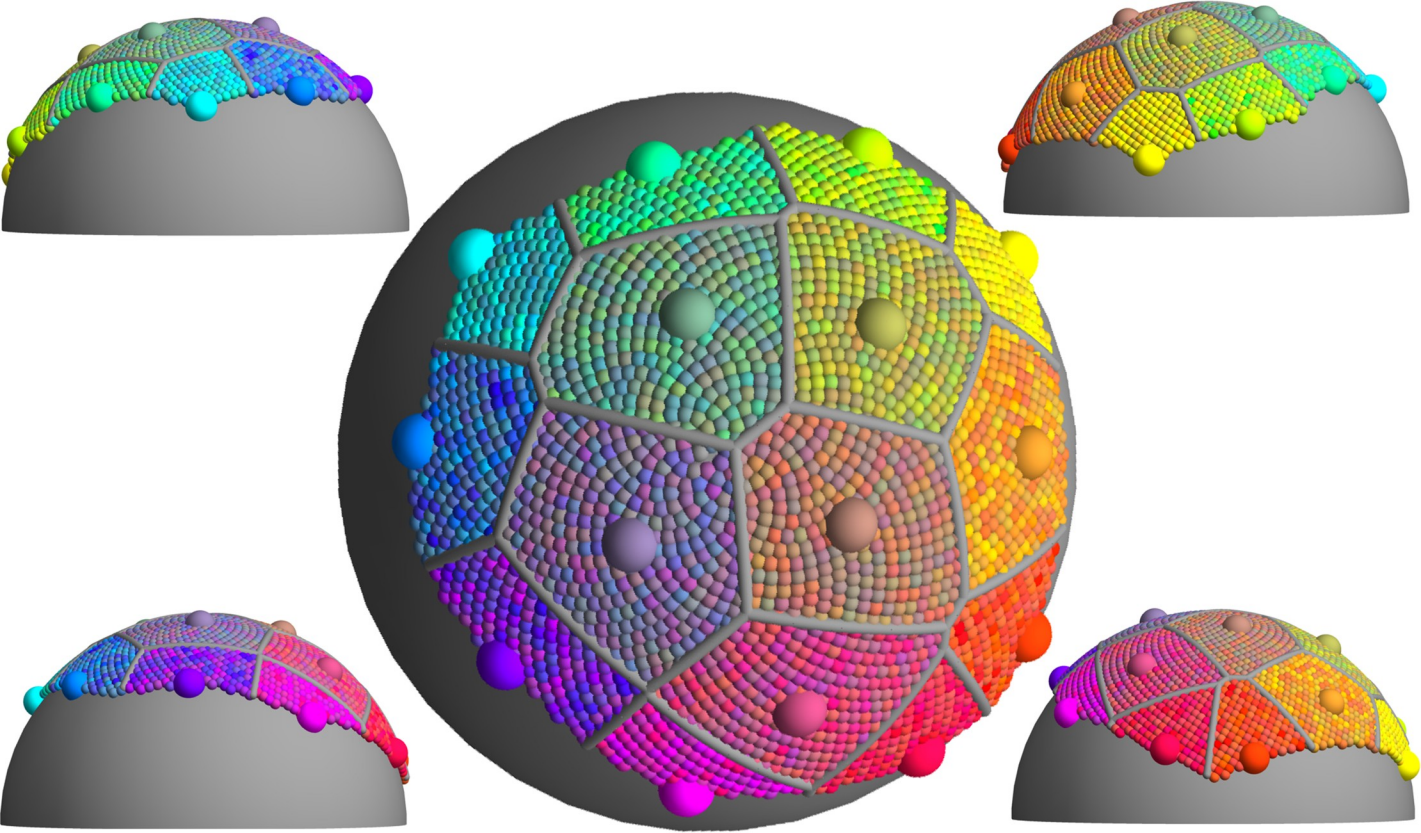
A Voronoi partition [78, 79] of the full colour surface (see Fig 14). The locations of the fifteen region centres (large spheres) were optimised to maximize nearest neighbour distances. All distances were measured within the approximately isometric embedding hence approximate geodesic categorical distances. Internal regions have area one areal-grain, boundary regions half an areal-grain. Region centres are coloured according to their locations, the colour of smaller spheres is randomly chosen from their containing region. The size of the regions is such that all colours within a region are categorically similar to the region centre, and all region centres are categorically distinct.

Now 2-D submanifolds. Hue triangles vary in their capacity. While the average capacity is 4.5, it ranges from 3.9 for bluish to 6.3 for reddish (much larger because of pinks and browns). Since the hue triangles have significant curvature along the boundaries, these capacities are likely under-estimates. The full colour surface has a capacity of 15.3, more than twice the largest hue triangle. We visualize this capacity in Fig 16.

Finally the full RGB cube has a capacity of 27.0, roughly twice that of the full colour surface. So, with a nod to many uncertainties we conclude that, for the subject population, the colour gamut can be partitioned into roughly 27 groups such that colours within a group would be considered categorically similar, and any two portions would be considered categorically distinct. Our count of 27 is similar to the results of some other studies, using different methods.

- Untrained subjects could, from the name, re-identify ~30 colours that they had previously named [31].
- Systematic colour naming systems with 29 names at level 2 [80], and 25 names at level 2 [81].
- Unconstrained colour naming of 200 colours by 50 subjects revealed 25 names used with high consistency [82].

but is lower than a previous estimate of 50 similarly based on inference from colour naming data [32].

## 8. Discussion

A colour geometry is a systematization of between-ness or distance relations between colours. Colour geometries have been sought to account for:

- psychophysical data (e.g. discrimination or matching thresholds) with practical applicability [16]
- behavioural data (e.g. comparison of large colour differences) that constrains theories of neural or cognitive representation.
- the phenomenology of colour [83]
- the logic of colour concepts [54]

Colour geometries, at the full level of detail of metric tensors throughout the colour solid, have previously been determined based on discrimination [84, 85] and matching [29]. We have presented the first colour geometry based on colour categorization, using Information Geometry to compute metric tensors in a principled way from population-level colour naming data.

Comparing the bases of the three geometries – discrimination, matching, categorization – we note some differences. The discrimination and matching tasks are based on comparison of a pair of colours, whereas the categorization task is based on one colour at a time. Discrimination and matching are feasible to study in single individuals, and similar results to a study based on multiple subjects would be expected; categorization would be much more difficult to study in a single individual, since excessive amounts of colour naming might well cause a change in behaviour, and the results would likely be different from the population-based geometry.

Focussing now on the categorical geometry, what are its uses? Practically it could be used as a metric for quantifying colour distortion in image reproduction media [86], penalizing greens turning into blues for example; or with image descriptors in computer vision, where there is evidence that colour categorical representations achieve good photometric invariance [87]; in interface design [88]; or in commercial applications such as product search-by-colour, or improved colour forecasting [89]. On the theory side, categorical geometry could be a new way to compare languages, to assess universality and deviations from it; and a new datum that begs explanation from culture, neurophysiology or environment. Pressing next steps of analysis are to compare the categorical geometry with the matching geometry [29], and to attempt an account of the categorical metric tensors as simple functions of cone responses, as per the original vision of Helmholtz [15] and Schrodinger [90].

We record some deficiencies in our analysis, which can stand as notes for any future study. 1. Better data collection: online is convenient but display variation will no doubt result in some blurring of estimated naming response functions. 2. Stimuli should be sampled randomly from sRGB cube rather than from a fixed set of colours. This would allow post hoc assessment of how much the colour sampling measure is leaking through to effect the estimated geometry. 3. The long tail of unseen naming responses should be modelled when estimating the geometry, not accounting for these may be biasing the estimate. 4. The similarity structure of names could be modelled and usefully fed into estimation of the geometry, at present *dark purple* is as different a response from *purple* as it is from *blue*, which does not seem quite right.

We end with a summary on the number of distinct colours according to different geometries. This subject is definitively reviewed in [29], which we acknowledge as an influential source for many aspects of the analysis and presentation in the current work. The discrimination, matching and categorical geometries differ in how many distinct colours (grains) they measure colour space to contain. In [29] previous discrimination-based counts were reviewed and a consensus estimate of 10^7^ grains was recommended, and new data was presented supporting a count of 1436 matching-based grains. The current paper counts 27 categorical grains. Given that colour space is three-dimensional, a digestible presentation of these numbers is as follows: 3^3^ categorical grains, each (on average) composed of 4^3^ matching grains, each (on average) composed of 20^3^ discrimination grains.
